# Cell constriction requires processive septal peptidoglycan synthase movement independent of FtsZ treadmilling in *Staphylococcus aureus*

**DOI:** 10.1038/s41564-024-01629-6

**Published:** 2024-03-13

**Authors:** Simon Schäper, António D. Brito, Bruno M. Saraiva, Georgia R. Squyres, Matthew J. Holmes, Ethan C. Garner, Zach Hensel, Ricardo Henriques, Mariana G. Pinho

**Affiliations:** 1 Instituto de Tecnologia Química e Biológica António Xavier, Universidade NOVA de Lisboa, Oeiras, Portugal; 2 Instituto Gulbenkian de Ciência, Oeiras, Portugal; 3 Department of Molecular and Cellular Biology, Harvard University, Cambridge, MA USA; 4 MRC-Laboratory for Molecular Cell Biology, University College London, London, UK

**Keywords:** Bacteriology, Cell division

## Abstract

Bacterial cell division requires recruitment of peptidoglycan (PG) synthases to the division site by the tubulin homologue, FtsZ. Septal PG synthases promote septum growth. FtsZ treadmilling is proposed to drive the processive movement of septal PG synthases and septal constriction in some bacteria; however, the precise mechanisms spatio-temporally regulating PG synthase movement and activity and FtsZ treadmilling are poorly understood. Here using single-molecule imaging of division proteins in the Gram-positive pathogen *Staphylococcus aureus*, we showed that the septal PG synthase complex FtsW/PBP1 and its putative activator protein, DivIB, move with similar velocity around the division site. Impairing FtsZ treadmilling did not affect FtsW or DivIB velocities or septum constriction rates. Contrarily, PG synthesis inhibition decelerated or stopped directional movement of FtsW and DivIB, and septum constriction. Our findings suggest that a single population of processively moving FtsW/PBP1 associated with DivIB drives cell constriction independently of FtsZ treadmilling in *S. aureus*.

## Abstract (web summary)

Single-molecule imaging reveals that peptidoglycan synthesis and synthase activity, rather than FtsZ treadmilling, are rate limiting and drive septum constriction in *Staphylococcus aureus*.

## Main

Bacterial cell division starts with mid-cell assembly of the divisome, a multi-protein complex spanning the membrane that is organized by the tubulin homologue FtsZ^1^. FtsZ monomers polymerize into protofilaments that form a dynamic and patchy polymer structure termed the Z-ring. This ring marks the future division site and acts as a scaffold to recruit other cell division proteins, including enzymes that synthesize peptidoglycan (PG), the main component of the bacterial cell wall^1^.

FtsZ has guanosine triphosphatase (GTPase) activity. Hydrolysis of GTP by FtsZ has a negative effect on the stability of protofilaments, leading to treadmilling behaviour around the division site^2–4^. Treadmilling is a motion of cytoskeletal filaments that grow in length at one end and simultaneously shrink at the opposite end, through the continuous addition and removal of protein subunits. Treadmilling FtsZ filaments condense into a dense Z-ring and initiate cell constriction by guiding septal cell wall synthesis^5,6^. Therefore, to trigger cell constriction, PG synthases must be spatio-temporally organized and their enzymatic activities tightly regulated^7–10^. While there is in vitro evidence supporting FtsZ-mediated membrane constriction^11^, PG synthesis is thought to be the main driving force for bacterial fission^12–14^.

PG synthesis requires the activity of glycosyltransferases (GTases), which polymerize glycan strands, and transpeptidases (TPases), which cross-link glycans via peptide bridges^15^. *Staphylococcus aureus* encodes a set of four penicillin-binding proteins (PBPs): class A PBP2, which combines GTase and TPase activities; class B PBP1 and PBP3, both with TPase activity only, with the septum-specific PBP1 having an essential function in cell division; and the low-molecular weight PBP4 with TPase activity^14,16–23^. Additionally, *S. aureus* contains two GTases from the shape, elongation, division and sporulation (SEDS) protein family^9,18,24,25^, FtsW and RodA. These proteins form cognate pairs with PBP1 and PBP3, and direct septal and lateral PG synthesis, respectively^18^.

The essential septal PG synthases FtsW/PBP1 are thought to be activated by a trimeric complex of the divisome proteins DivIB, DivIC and FtsL (named FtsQ, FtsB and FtsL, respectively, in Gram-negative bacteria), which are conserved in cell wall-producing bacteria^26–29^. The DivIB/DivIC/FtsL subcomplex is required to recruit MurJ, the flippase of the PG precursor lipid II, to the division site, driving PG incorporation to mid-cell^14^. Arrival of MurJ marks the transition between two stages of cytokinesis, as FtsZ treadmilling is essential for cell division at the initiation of constriction, before MurJ arrival, but becomes dispensable for septum synthesis afterwards^14^.

Recent studies have indicated differences among bacterial species regarding the role of FtsZ treadmilling in cell division and in the spatio-temporal distribution of septal PG synthases. In *Escherichia coli*, FtsZ treadmilling directs the processive movement of a fast subpopulation of non-active septal PG synthases, presumably to ensure their homogeneous distribution around the division site, but does not limit the rate of septum closure^4,30^. Interestingly, active PG synthases move with a slower velocity, independently of FtsZ treadmilling, suggesting a two-track model for active and inactive PG synthases in *E. coli*^30^. In contrast, the rate of FtsZ treadmilling in *Bacillus subtilis* correlates both with the velocity of septal PG synthases and the rate of cell division^3^. FtsZ treadmilling in this organism is essential for guiding septal cell wall synthesis during constriction initiation, after which it becomes dispensable for division, while maintaining a role in accelerating the septum constriction rate^5^. On the other hand, in *Streptococcus pneumoniae*, neither the rate of septum synthesis nor the velocity of the septal PG synthases is determined by treadmilling of FtsZ filaments^31^. Although these models exhibit overlapping trends, they raise questions about the conservation of mechanisms that control septal PG synthesis in bacteria.

In this Article, we analysed movement dynamics of key proteins involved in Z-ring assembly and septal PG synthesis during *S. aureus* cell division. Using single-molecule tracking, we describe the directional movement of FtsW, PBP1 and DivIB around the division site, each existing in a single motile population, and show that their velocities are dependent on septal PG synthesis and independent of FtsZ treadmilling. We propose a one-track model for septal PG synthases, featuring FtsZ treadmilling-independent movement of active FtsW/PBP1 complexes, in association with DivIB, to drive the synthesis of the new septum.

## Results

### Septal PG synthesis is rate limiting for cell constriction

We have previously shown that cells of the coccoid bacterium *S. aureus* treated with the FtsZ inhibitor PC190723 fail to initiate synthesis of a new division septum, but do not stop ongoing constriction of existing septa, suggesting that FtsZ treadmilling is required only during the initial stage of cytokinesis^14^. Similarly, FtsZ treadmilling in rod-shaped *B. subtilis* is essential for constriction initiation but becomes dispensable after the arrival of PG synthesis machinery, although it remains rate limiting for cell division^5^. We set out to determine whether the rate of septum constriction is accelerated by FtsZ treadmilling in *S. aureus* as well. For this, we constructed mutants with slowed FtsZ treadmilling in methicillin-resistant *S. aureus* strains COL (hospital associated) and JE2 (community associated) by replacing their native *ftsZ* gene with an allele encoding the GTPase point mutation T111A. The same amino acid substitution in *B. subtilis* FtsZ caused an approximately tenfold reduction in GTPase activity^32^. The thermosensitive FtsZ(T111A) mutants in COL and JE2 backgrounds produced FtsZ at wild-type levels (Supplementary Fig. 1a) and incorporated fluorescent d-amino acid HCC-amino-d-alanine (HADA)^33^ into septal PG (Supplementary Fig. 1b). Furthermore, the FtsZ(T111A) mutation in the COL strain did not impair normal cell cycle progression at 30 °C (Supplementary Fig. 2a,c). However, the mutant showed heterogeneous cell size (Supplementary Fig. 2a,e) and growth rates of mutant derivative strains of COL and JE2 at 30 °C were reduced by 6% and 16%, respectively (Supplementary Fig. 3a). The growth defect of strain JE2 FtsZ(T111A) was accompanied by morphological defects, as cells grown at 37 °C displayed multiple and misplaced septa (Supplementary Fig. 4).

**Fig. 1:**
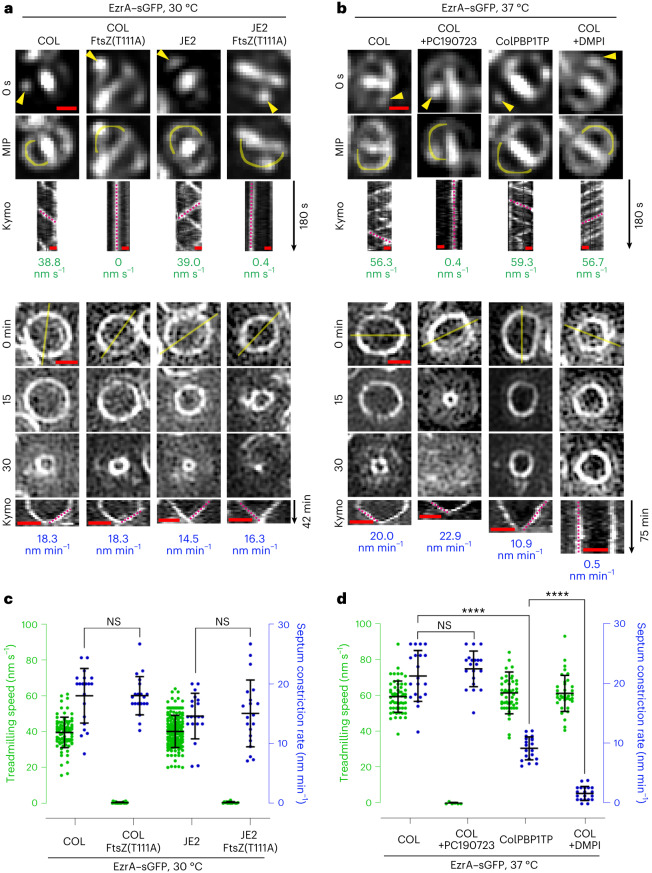
Septum constriction is decelerated in cells lacking PBP1 TPase activity but not in cells with impaired FtsZ treadmilling. **a**,**b**, Representative fluorescence micrographs of EzrA–sGFP in COL and JE2 backgrounds producing either FtsZ wild type or thermosensitive FtsZ(T111A) mutant variants at 30 °C (**a**), or in PBP1 TPase mutant ColPBP1TP and COL backgrounds at 37 °C in the absence and presence of FtsZ inhibitor PC190723 or MurJ inhibitor DMPI (**b**). Top: epifluorescence images of EzrA–sGFP in late pre-divisional cells with nascent Z-rings at the start (0 s) and throughout a 180-s time series (maximum intensity projection, MIP). Yellow arrow heads indicate EzrA–sGFP patches whose localization was followed over time. Space–time kymographs were generated by extracting fluorescence intensity values along indicated yellow freehand lines. Magenta dashed straight lines indicate slopes used to calculate EzrA–sGFP speed (nm s^−1^), a proxy for FtsZ treadmilling speed. Bottom: structured illumination microscopy images of constricting EzrA–sGFP rings. Magenta dashed straight lines in kymographs indicate slopes used to calculate septum constriction rates (nm min^−1^). Scale bars, 0.5 µm. **c**,**d**, Scattered dot plots of EzrA–sGFP speed (green) and Z-ring constriction rates (blue). Bars indicate means and standard deviations of slopes obtained from lines drawn on both types of kymographs of COL EzrA–sGFP (*n*_1_ = 83 and *n*_2_ = 20), COL EzrA–sGFP FtsZ(T111A) (*n*_1_ = 21 and *n*_2_ = 20), JE2 EzrA–sGFP (*n*_1_ = 171 and *n*_2_ = 20;) and JE2 EzrA–sGFP FtsZ(T111A) (*n*_1_ = 15 and *n*_2_ = 20) (**c**), and COL EzrA–sGFP (*n*_1_ = 56 and *n*_2_ = 20), COL EzrA–sGFP+PC190723 (*n*_1_ = 6 and *n*_2_ = 20), ColPBP1TP EzrA–sGFP (*n*_1_ = 51 and *n*_2_ = 20) and COL EzrA–sGFP+DMPI (*n*_1_ = 37 and *n*_2_ = 20) (**d**). *n*_1_, number of analysed slopes obtained from EzrA–sGFP movement kymographs. *n*_2_, number of analysed slopes obtained from EzrA–sGFP ring constriction kymographs of independent cells. Data were obtained from two independent biological replicates. Statistical analysis was performed using a two-tailed Mann–Whitney *U* test. n.s., not significant (*P* ≥ 0.05). ****P < 0.0001. ColPBP1TP EzrA–sGFP versus COL EzrA–sGFP (*P* = 5.8 × 10^−11^). COL EzrA–sGFP+DMPI versus ColPBP1TP EzrA–sGFP (*P* = 1.5 × 10^−11^). Source data

To determine FtsZ treadmilling speed in nascent Z-rings, we used, as a proxy, a functional fusion of EzrA to superfast green fluorescent protein (sGFP). EzrA is a direct interaction partner of FtsZ, with similar movement dynamics, sensitive to FtsZ-targeting PC190723^14^. The movement of EzrA–sGFP patches around new division sites was visualized by time-resolved microscopy (acquiring images every 3 s) and their spatial position over time was plotted in kymographs. As expected, the speed of EzrA–sGFP movement (from here on referred to as FtsZ treadmilling) in the FtsZ(T111A) mutants was severely reduced relative to the parental strains (Fig. 1a,c, Supplementary Table 1 and Supplementary Video 1). EzrA–sGFP levels were not affected by the FtsZ(T111A) mutation (Supplementary Fig. 1a). To measure the rate of septum constriction in dividing *S. aureus* cells, we imaged EzrA–sGFP rings (acquiring images every 3 min) and plotted fluorescence intensity along the cell diameter in space–time kymographs. Surprisingly, despite nearly complete inhibition of FtsZ treadmilling and the presence of cells with spiralling septa in FtsZ(T111A) mutants (Supplementary Video 2), septum constriction rates were remarkably similar to those of the respective parental strains (Fig. 1a,c, Supplementary Table 1 and Supplementary Video 2). Likewise, treating COL cells with PC190723^34^ to inhibit FtsZ treadmilling did not significantly affect the septum constriction rate (Fig. 1b,d, Supplementary Table 1 and Supplementary Videos 3 and 4). These results indicate that, unlike in *B. subtilis*, GTPase-driven FtsZ treadmilling is not rate limiting for septum constriction in *S. aureus*.

Next we analysed septum constriction in a COL mutant lacking TPase activity of the division-specific PG synthase PBP1 (ColPBP1TP), which results in decreased PG cross-linking^18^. Our attempts to generate the same mutation in the JE2 background were unsuccessful. The growth rate of ColPBP1TP remained unaffected (Supplementary Fig. 3b). However, septum constriction rate was reduced ~twofold (Fig. 1b,d, Supplementary Table 1 and Supplementary Video 4) and, in agreement, cell cycle analysis revealed a larger fraction of ColPBP1TP cells with a constricting septum than for the parental strain COL (Supplementary Fig. 2b,d,f), in line with a previous report showing a septum completion defect in PBP1-depleted cells^16^. Furthermore, treatment of COL cells with 3-{1-[(2,3-dimethylphenyl) methyl]piperidin-4-yl}-1-methyl-2-pyridin-4-yl-1H-indole (DMPI)^35^, an inhibitor of the lipid II flippase MurJ, that blocks PG synthesis, almost completely halted septum constriction (Fig. 1b,d, Supplementary Table 1 and Supplementary Video 4), in accordance with our previous data^14^. Importantly, in both conditions where the septum constriction rate was slowed down, FtsZ treadmilling speed remained unchanged (Fig. 1b,d, Supplementary Table 1 and Supplementary Video 3). These results show that septal PG synthesis (resulting from FtsW/PBP1 activity), not FtsZ treadmilling, is the primary driver of cell constriction in *S. aureus*.

### FtsW, PBP1 and DivIB move directionally around the division site

**Fig. 2:**
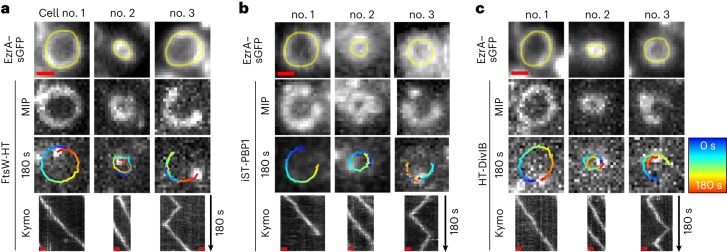
FtsW, PBP1 and DivIB move directionally around the division site. **a**–**c**, Representative epifluorescence micrographs of cells of JE2 EzrA–sGFP producing FtsW-HT (**a**), iST-PBP1 (**b**) or HT-DivIB (**c**), and grown in TSB rich medium at 37 °C. Cells were sparsely labelled with the fluorescent ligands JF549-HTL or JFX650-STL to visualize single molecules of FtsW-HT and HT-DivIB or iST-PBP1, respectively. Each panel shows three independent cells, of which no. 1 and no. 2 are at an early and a late stage of septum constriction, respectively, and no. 3 exhibits a labelled molecule transitioning between clockwise and counter-clockwise movement around the division site. Single-molecule images acquired in the last frame of a 180-s time series are overlayed with tracks, where blue (0 s) to red (180 s) indicates trajectory time. Space–time kymographs were generated by extracting fluorescence intensity values from FtsW-HT, iST-PBP1 and HT-DivIB images along yellow freehand lines drawn on corresponding EzrA–sGFP rings acquired with the last frame of each time series. MIP, maximum intensity projection, of all 61 time frames. Scale bars, 0.5 µm.

Our data indicate that PBP1 TPase activity is a major driver of septum constriction. In *S. aureus*, PBP1 acts in concert with its cognate SEDS family protein FtsW^18^. The homologous division SEDS–bPBP pairs in *B. subtilis*, *S. pneumoniae*, *E. coli* and *Caulobacter crescentus* undergo directional movement at the division site^6,30,31,36^. We aimed to investigate whether FtsW/PBP1, and other components of the cell division machinery, show similar movement behaviour in *S. aureus* cells. For this, we constructed self-labelling Halo-tag (HT)^37^ and Snap-tag (ST)^38^ fusions to various cell division and cell wall-related proteins in the background of strain JE2. Genomic *ftsW*, *murJ*, *pbp4* and *gpsB* were replaced with gene derivatives encoding C-terminal translational fusions to HT. Strains expressing *ht* fused to *ftsW* and *murJ* genes from the native locus, under the control of their native promoter, as the sole copy of the gene, were viable, indicating functionality of the corresponding protein fusions, given that both genes are essential in methicillin-resistant *S. aureus*^14^. Expression of gene fusions inserted at the ectopic *spa* locus was driven either by the isopropyl β-d-1-thiogalactopyranoside (IPTG)-inducible *spac* promoter (*ht-divIB* and *rodA-ht*) or by the anhydrotetracycline (Atc)-inducible *xyl-tetO* promoter (*ist-pbp1*, *ftsZ-ht* and *ezrA-ht*). Our attempts to construct functional fusions to *pbp2* and *pbp3* were unsuccessful. Production of the protein fusions did not affect *S. aureus* growth, except for GpsB-HT (Supplementary Figs. 5a and 6a). Moreover, HT and ST protein fusions were not proteolytically cleaved and were enriched at the septum as expected (Supplementary Fig. 5b,c), except for iST-PBP1, which showed additional cytoplasmic fluorescence probably attributed to partial proteolytic cleavage of the protein fusion (Supplementary Fig. 6b,c). We then studied by single-molecule tracking microscopy (acquiring images every 3 s) the movement dynamics of the nine functional HT and ST fusions at the division site, which was labelled with early cell division protein EzrA fused to sGFP. To resolve single molecules of HT and ST protein fusions, exponentially growing cells were sparsely labelled with JF549-HTL and JFX650-STL, respectively. Directional movement around the division site was observed for single molecules of FtsW-HT, iST-PBP1 and HT-DivIB, in both early and late stages of septum constriction (Fig. 2, Supplementary Table 2 and Supplementary Video 5). In some cases, the travelling distance of molecules could span the entire septum circumference during the 180-s observation period, corresponding to a track length of ~3 μm, and a fraction of molecules transitioned between clockwise and counter-clockwise circumferential movement (Fig. 2). We also tested a DivIB variant lacking its C-terminal residues 373–439, given that an equivalent mutant of *E. coli* FtsQ (DivIB orthologue) failed to interact with other divisome proteins and impeded their recruitment to mid-cell^39,40^. Interestingly, this truncation abolished directional movement of HT-DivIB (Supplementary Table 2) and caused mis-localization (Supplementary Fig. 7a), but did not notably affect the fusion’s stability (Supplementary Fig. 7e). No directional movement around the division site was observed for single molecules of MurJ-HT, PBP4-HT, GpsB-HT, RodA-HT, FtsZ-HT and EzrA-HT (Supplementary Table 2). The movement of these molecules was not analysed further due to a lack of continuous tracks with at least 30 data points and that were above the cut-off for track directionality (*α*_MSD_ ≥ 1) (Supplementary Table 2). We concluded that divisome proteins have distinct movement dynamics at the division site, with FtsW, PBP1 and DivIB moving directionally.

### FtsW, PBP1 and DivIB move with the same velocity

**Fig. 3:**
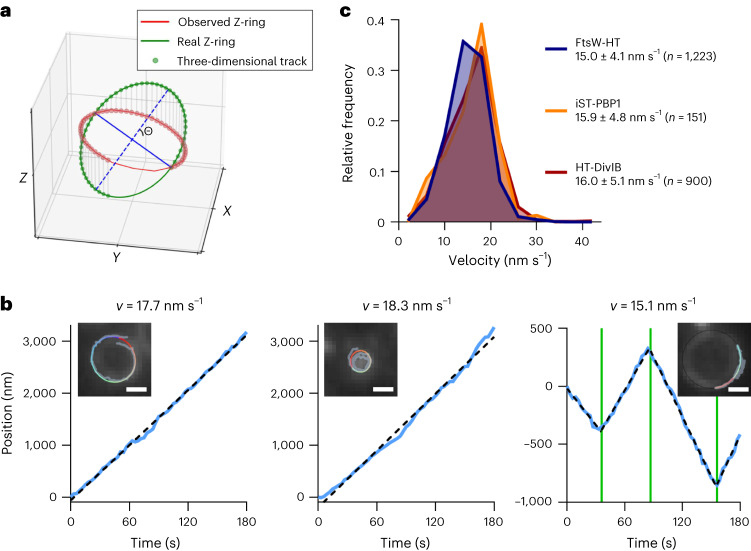
FtsW, PBP1 and DivIB move directionally with the same velocity. **a**, Representation of a simulated single-molecule track of FtsW-HT aligned to an ellipse that was drawn on top of an observed EzrA–sGFP ring. *Θ* indicates the angle between the imaging and division planes (Supplementary Video 6). **b**, Examples of unwrapped FtsW-HT trajectories (blue lines in graphs). Left and middle graphs show FtsW-HT in cells at an early and a late stage of septum constriction, respectively, and the right graph shows FtsW-HT transitioning between different directions. Green vertical lines indicate detected breakpoints used to segment tracks to perform a linear regression in each section (indicated by black dashed lines). Average velocity (*v*) was calculated as the mean of velocities determined for each section. Insets show corresponding micrographs of EzrA–sGFP overlayed with the original track (grey) and the same track after alignment with an ellipse (blue to red indicates trajectory time). Scale bars, 0.5 µm. **c**, Histograms of FtsW-HT, iST-PBP1 and HT-DivIB single-molecule velocities determined in JE2 EzrA–sGFP background in TSB rich medium at 37 °C. Average velocity is shown as mean with standard deviation. Bin width, 4. Centre of first/last bin, 2/42. Histograms were obtained from at least six biological replicates. Source data

To characterize the directional movement of FtsW, PBP1 and DivIB molecules, we started by determining their velocity. *S. aureus* cells are approximately spherical and, when placed on a microscope slide, will present the division plane in random orientations. Therefore, our analysis considered single-molecule motion in all three spatial dimensions by making two assumptions: (1) directionally moving molecules move on division rings and (2) the cytokinetic ring has a near-circular shape. Briefly, the molecules’ *Z*-position (position along the axis perpendicular to the imaging plane) was inferred by aligning their *X*–*Y* position with the nearest point in an ellipse manually drawn on a corresponding EzrA–sGFP ring, and rotating this ellipse by $\Theta$ (angle of the division ring relative to the imaging plane) into a circle (Fig. 3a and Supplementary Video 6). To estimate the velocity of single molecules, we analytically unwrapped each track into a one-dimensional representation. Tracks were then sectioned using breakpoint detection, velocity was calculated on the basis of simple linear regression for each section and the average velocity of the sections of each track was determined (Fig. 3b). Following this approach, consistent molecule velocities were obtained over a range of $\Theta$ from 0° to ~75° (Supplementary Fig. 8a–c). We noticed that tracks in cells with a division plane nearly perpendicular ($\Theta$ > 75°) to the imaging plane resulted in larger standard deviations of calculated velocities due to frequent unwrapping artefacts and, for this reason, only tracks with $\Theta$ < 75° were included in further analysis.

In JE2 and COL strains, the average velocities of FtsW-HT, iST-PBP1 and HT-DivIB, directionally moving for at least 87 s (equivalent to 30 data points in a track), were similar (~15–16 nm s^−1^) (Fig. 3c and Supplementary Table 1). Moreover we found no correlation between their average velocity and either track duration or cell division stage (Supplementary Fig. 8d–i). The similar velocities observed for FtsW, PBP1 and DivIB throughout all stages of constriction suggest that these proteins exist in a complex, which moves at a constant rate from initiation to completion of septum constriction.

### FtsW, PBP1 and DivIB velocity correlates with cell growth rate

**Fig. 4:**
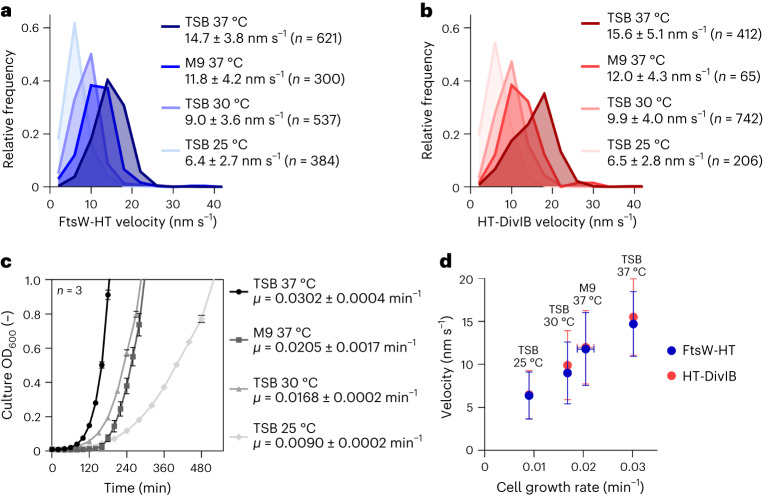
FtsW and DivIB velocity correlates with cell growth rate. **a**,**b**, Histograms of FtsW-HT (**a**) and HT-DivIB (**b**) single-molecule velocities determined in JE2 EzrA–sGFP background, in rich and poor media and at various growth temperatures. Average velocity is shown as mean with standard deviation. Bin width, 4. Centre of first/last bin, 2/42. **c**, Mean optical density of culture as a function of time, determined for strain JE2 EzrA–sGFP grown in indicated media and at various temperatures. Error bars represent the standard deviations of three biological replicates. **d**, FtsW-HT and HT-DivIB mean velocities shown in **a** and **b** as a function of cell growth rate determined from growth curves shown in **c**. Horizontal error bars represent the standard deviations from three biological replicates and vertical error bars represent the standard deviations of a minimum of 65 trajectories from three biological replicates. Source data

Having established a quantitative approach to determine their velocity, we analysed directionally moving molecules of FtsW and DivIB in fast and slow growth conditions. From here on, we used FtsW velocity as a proxy for that of PBP1 because both proteins were previously shown to directly interact at the division site^18^. FtsW-HT and HT-DivIB average velocities in JE2 cells grown in M9 minimal medium were reduced ~1.3-fold relative to cells grown in tryptic soy broth (TSB) rich medium (Fig. 4a,b and Supplementary Table 3). Average velocities of the same proteins in TSB rich medium were reduced ~1.6-fold when the growth temperature was reduced from 37 °C to 30 °C, and ~2.3-fold when the temperature was decreased from 37 °C to 25 °C (Fig. 4a,b and Supplementary Table 3). Cellular levels of FtsW-HT, PBP1 and EzrA–sGFP remained unchanged in the different growth conditions (Supplementary Fig. 9), suggesting that the number of divisome complexes was the same in cells with varying FtsW and DivIB velocities. As expected, cell growth rate was slower in M9 minimal medium or at lower temperatures than in TSB rich medium at 37 °C (Fig. 4c). Plotting FtsW-HT and HT-DivIB average velocity as a function of cell growth rate indicated a positive linear correlation between these two parameters (Fig. 4d).

### FtsW and DivIB movement does not depend on FtsZ treadmilling

**Fig. 5:**
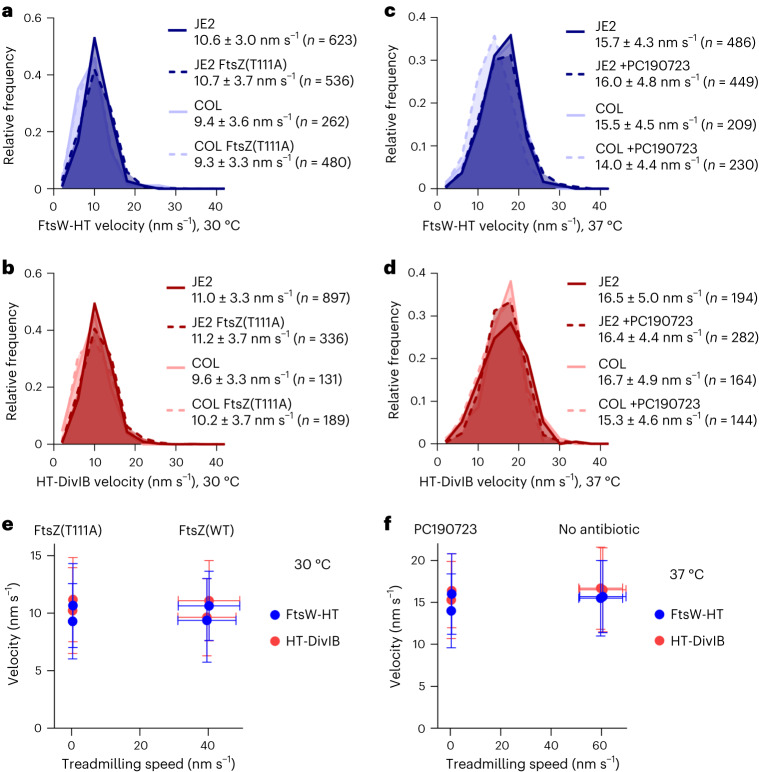
FtsW and DivIB velocity does not correlate with FtsZ treadmilling speed. **a**–**d**, Histograms of FtsW-HT (**a** and **c**) and HT-DivIB (**b** and **d**) single-molecule velocities determined in JE2 EzrA–sGFP and COL EzrA–sGFP backgrounds, each producing either FtsZ wild type or temperature-sensitive FtsZ GTPase mutant T111A, from its native genomic locus, in TSB rich medium at 30 °C (**a** and **b**), or in the absence and presence of FtsZ inhibitor PC190723 in TSB rich medium at 37 °C (**c** and **d**). Average velocity is shown as mean with standard deviation. Bin width, 4. Centre of first/last bin, 2/42. **e**,**f**, FtsW-HT and HT-DivIB mean velocities shown in **a**,**b** (**e**) and **c**,**d** (**f**) as a function of FtsZ treadmilling speed shown in Fig. 1 and Supplementary Table 1. Horizontal error bars represent the standard deviations of a minimum of 6 and a maximum of 171 slopes and vertical error bars represent the standard deviations of a minimum of 131 trajectories from three biological replicates. Source data

The directional movement of septum-specific PG synthases can be dependent or independent of treadmilling FtsZ filaments, depending on the species^3,4,30,31^. To examine whether FtsW and DivIB velocity correlates with FtsZ treadmilling speed in *S. aureus*, we first used the FtsZ(T111A) mutants. This mutation, which did not affect FtsW-HT co-localization with EzrA–sGFP at the division site (Supplementary Fig. 1b), severely reduced FtsZ treadmilling speeds in both JE2 and COL backgrounds (Fig. 1a,c). Despite this dramatic decrease, FtsW-HT and HT-DivIB velocities were not markedly changed (Fig. 5a,b, Supplementary Tables 1 and 3 and Supplementary Video 7). Similarly, PC190723 treatment of cells, which virtually stopped FtsZ treadmilling (Fig. 1b,d), did not affect FtsW-HT or HT-DivIB velocities (Fig. 5c,d, Supplementary Tables 1 and 3 and Supplementary Video 5). Importantly, FtsW-HT and HT-DivIB average velocities were not affected by impaired FtsZ treadmilling at any stage of cytokinesis, even at the initial stage during which FtsZ treadmilling is essential^14^ (Supplementary Fig. 10). Moreover, FtsW-HT and HT-DivIB velocities in JE2 and COL backgrounds, at 30 °C and 37 °C, were ~fourfold slower than FtsZ treadmilling speeds at the respective growth temperature (Supplementary Table 1), indicating that FtsW and DivIB do not move together with FtsZ. Combined, these results indicate that the movement of *S. aureus* FtsW and DivIB along septal rings is not coupled to FtsZ treadmilling (Fig. 5e,f).

### Septal PG synthesis drives FtsW/PBP1 and DivIB movement

**Fig. 6:**
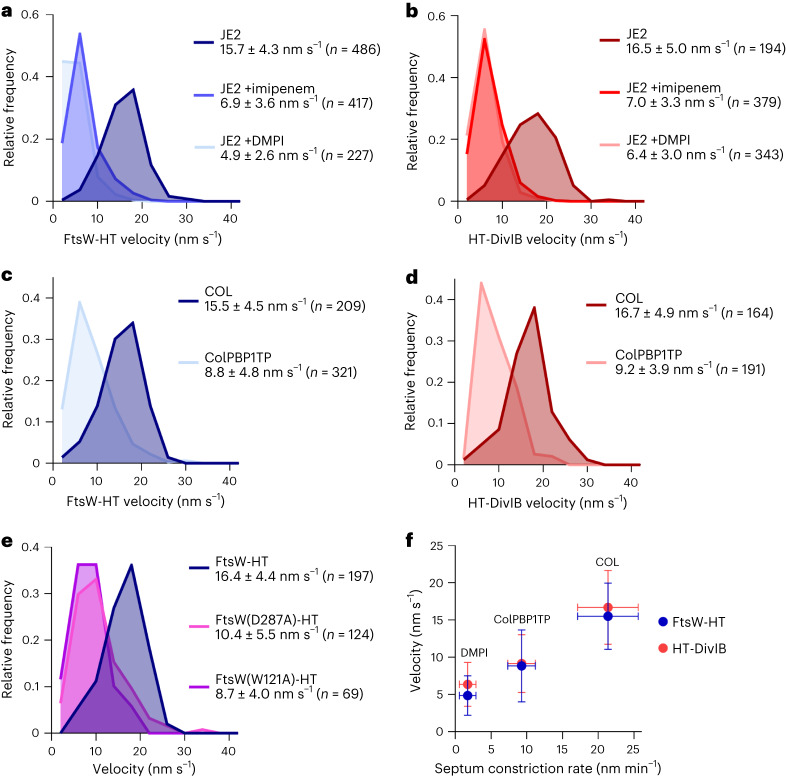
Inhibition of PG synthesis slows down the directional movement of FtsW and DivIB. **a**–**e**, Histograms of FtsW-HT (**a**, **c** and **e**) and HT-DivIB (**b** and **d**) single-molecule velocities determined in JE2 EzrA–sGFP and COL EzrA–sGFP backgrounds in the absence and presence of β-lactam imipenem or MurJ inhibitor DMPI (**a** and **b**), in PBP1 TPase mutant ColPBP1TP EzrA–sGFP and COL EzrA–sGFP backgrounds (**c** and **d**), and in JE2 EzrA–sGFP background producing either FtsW-HT wild type or its active-site mutant derivatives W121A and D287A from the ectopic *spa* locus (**e**). Strains were grown in TSB rich medium at 37 °C. Average velocity is shown as mean with standard deviation. Bin width, 4. Centre of first/last bin, 2/42. **f**, FtsW-HT and HT-DivIB mean velocities shown in **a,c** and **b,d**, respectively, as a function of septum constriction rate shown in Fig. 1 and Supplementary Table 1. Horizontal error bars represent the standard deviations of 20 cells and vertical error bars represent the standard deviations of a minimum of 164 trajectories from three biological replicates. Source data

Given that FtsZ treadmilling was not the driver for the directional movement of FtsW/PBP1 and DivIB in *S. aureus*, we next asked whether their velocity was determined by PG synthesis. Treatment of cells with DMPI^35^, an inhibitor of the lipid II flippase MurJ, did not slow down FtsZ treadmilling speed (Fig. 1b,d), but caused a ~threefold reduction in FtsW-HT and HT-DivIB average velocities (Fig. 6a,b, Supplementary Tables 1 and 3 and Supplementary Video 5). Treatment of cells with imipenem, a β-lactam with preferential activity against *S. aureus* PBP1^41^, also resulted in a twofold reduction in FtsW-HT and HT-DivIB average velocities (Fig. 6a,b, Supplementary Table 3 and Supplementary Video 5). Strikingly, treatment of cells with the glycopeptide antibiotic vancomycin that inhibits PG synthesis, previously shown to cause total arrest of *S. aureus* cell division at all stages within minutes^42^, nearly completely stopped the directional movement of FtsW-HT and HT-DivIB (Supplementary Fig. 11 and Supplementary Video 5). The frequency of cells exhibiting directionally moving molecules was reduced from ~6-8% in untreated cells to ~0.1-0.2% in vancomycin-treated cells (Supplementary Table 3). Noteworthy, vancomycin treatment did not affect FtsW-HT or EzrA–sGFP localization to mid-cell, suggesting that divisomes remained intact (Supplementary Fig. 7b).

We then used active-site FtsW GTase or PBP1 TPase mutants to test which PG synthesis activity was required for the processive movement of the two proteins. Inactivation of PBP1 TPase activity via a S314A mutation in the ColPBP1TP strain decreased FtsW-HT and HT-DivIB velocities by ~1.5–2-fold relative to the parental COL strain (Fig. 6c,d, Supplementary Table 3 and Supplementary Video 8), while track duration and FtsW-HT localization to mid-cell remained unchanged (Supplementary Fig. 7c and Supplementary Table 3). Next, the GTase point mutations W121A and D287A, previously shown to impair the essential function of FtsW in *S. aureus*^18^, were introduced into the FtsW-HT fusion produced from the ectopic *spa* locus. As a control, we introduced wild-type FtsW-HT into the *spa* locus and confirmed that its velocity was the same when produced either ectopically, from the *spa* locus, or from its native locus (Fig. 6a,e). Relative to wild-type FtsW-HT, the velocities of the FtsW GTase mutants were reduced between ~1.5- and ~2-fold, while all ectopically produced protein variants localized to mid-cell and were subject to no apparent proteolytic cleavage (Fig. 6e and Supplementary Fig. 7d,e). Furthermore, slowed velocities of FtsW GTase mutant variants were accompanied by reductions in the percentage of cells with tracks from ~3.8% to ~0.4–0.9% (Supplementary Table 3). These results indicate that the processive movement of FtsW/PBP1 and DivIB along septal rings is driven by FtsW GTase and/or PBP1 TPase enzyme activities.

### FtsW and DivIB exist in a single motile population

Previous studies using Gram-negative *E. coli* and *C. crescentus* proposed a two-track model where directionally moving FtsW exists in two motile populations, one slow (active) depending on PG synthesis and one fast (inactive) depending on FtsZ treadmilling^30,36^. To test if this was also the case for *S. aureus*, we analysed FtsW and DivIB dynamics without averaging the velocities of each track. This allows detection of molecules that would switch between slow and fast movement, giving rise to bimodal distributions. FtsW-HT velocities showed a unimodal distribution (Supplementary Fig. S12). In *E. coli*, depletion of the slow (active) subpopulation of FtsW through inhibition of PG synthesis, leads to an increase of the overall average speed of FtsW molecules^30^. In *S. aureus*, we observed a decrease of FtsW-HT and HT-DivIB velocities and no appearance of a new, fast population of FtsW-HT molecules when slowing down the cell growth rate, inhibiting PG synthesis by DMPI treatment or inactivating PBP1 TPase and FtsW GTase (Supplementary Fig. 12c–g). Furthermore, the unimodal distribution of FtsW-HT velocity was still observed using three times faster image acquisition rate (increase from 0.33 Hz to 1 Hz) (Supplementary Fig. 12h). Combined with our finding that inhibition of PG synthesis by vancomycin stopped the directional movement of FtsW and DivIB, these data suggest that these two proteins exist in a single motile population dependent on PG synthesis.

### FtsW/DivIB velocity correlates with septum constriction rate

**Extended Data Fig. 1:**
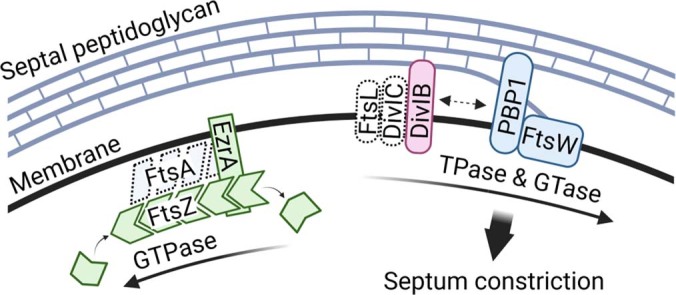
Model for the movement dynamics of essential cell division proteins in *S. aureus*. The directional movement of the septal peptidoglycan synthases FtsW and PBP1 is driven by their glycosyltransferase (GTase) and/or transpeptidase (TPase) activities. Processively moving FtsW/PBP1 complexes remain associated with the activating sub-complex DivIB/DivIC/FtsL to synthesize new peptidoglycan and thereby promote inward growth of the division septum. In this model, GTPase-driven treadmilling by FtsZ filaments neither determines the velocity of the essential PG synthesis enzyme complex, nor the rate of septum synthesis in *S. aureus*. Note that FtsA, DivIC and FtsL movement dynamics were not analysed in this study.

As PBP1 TPase and/or FtsW GTase activities were required for directional movement of FtsW (Fig. 6c,e) and were rate limiting for septum constriction (Fig. 1b,d), we hypothesized that the rates of septal PG synthesis and septum constriction may correlate. Plotting the average velocities of FtsW-HT and HT-DivIB as a function of septum constriction rate, using data points obtained for COL, ColPBP1TP mutant lacking PBP1 TPase activity and cells treated with MurJ inhibitor DMPI, revealed a positive linear correlation between these two parameters (Fig. 6f). This data supports a model in which the enzymatic activities by FtsW/PBP1 complexes, in concert with DivIB, determine the rate of septal PG synthesis and hence the rate of septum synthesis in *S. aureus* (Extended Data Fig. 1).

## Discussion

In this work, we examined the movement dynamics of cell division proteins arriving early (FtsZ and EzrA) or late (FtsW, PBP1, DivIB, GpsB and MurJ) at the division site of *S. aureus*, as well as the movement dynamics of the elongasome protein RodA and the PG TPase PBP4. Single-molecule imaging robustly detected directional movement around the division plane only for FtsW, PBP1 and DivIB. Consistent with previous data, FtsZ and EzrA move around the division site in treadmilling patches/filaments, rather than as single molecules^5,6,14^. A previous study of *B. subtilis* revealed two dynamically distinct subcomplexes of the divisome, one composed of stationary FtsZ binding proteins (FtsA, EzrA, SepF and ZapA) and one containing directionally moving cell wall synthases (FtsW, PBP2B, DivIB, DivIC and FtsL)^6^. Given that in our study FtsW/PBP1 and DivIB showed the same directional movement in all conditions tested, we hypothesize that *S. aureus* FtsW/PBP1 engage in a stable complex with the DivIB/DivIC/FtsL subcomplex. This hypothesis is in agreement with a recent study describing the isolation of the *Pseudomonas aeruginosa* septal PG synthesis enzyme complex comprising the proteins FtsQ (DivIB orthologue), FtsB (DivIC orthologue), FtsL, FtsW and FtsI (orthologue of *S. aureus* PBP1)^43^. The predicted structure of the orthologous complex in *S. aureus* (Supplementary Fig. 13) is similar to that reported for Gram-negative bacteria^43,44^. In this model, the extracytoplasmic DivIB β and γ domains seem to play a role in mediating the predicted interaction between DivIB and the PBP1 PASTA domains, in agreement with a reported interaction between the *B. subtilis* homologues^45^. We have previously shown that DivIB/DivIC/FtsL is required to recruit the lipid II flippase MurJ^14^, thereby setting on septal PG incorporation. Since we were unable to robustly detect directional movement of a functional MurJ fusion, our data suggest that in *S. aureus* MurJ does not stably interact with a putative pentameric complex of FtsW, PBP1, DivIB, DivIC and FtsL.

Our findings indicate that FtsW/PBP1 velocity scales with constriction rate and remains constant at all stages of cytokinesis, suggesting that, once initiated, cell constriction in *S. aureus* is a continuous process driven by PG synthesis. In our experiments, PG synthesis was inhibited either by using active-site mutants of FtsW and PBP1, or by treating cells with antibiotics targeting different steps in the PG synthesis pathway. In all cases, FtsWʹs directional movement was either slowed down or completely stopped, suggesting that directionally moving FtsW/PBP1 complexes in *S. aureus* are enzymatically active (Extended Data Fig. 1). Based on the strict dependence of FtsW movement on septal PG synthesis, we hypothesize that tracks spanning (almost) the entire circumference of the division site may correspond to the synthesis of long glycan strands that are later cleaved by PG hydrolases. Similarly, it is possible that the FtsW/PBP1 molecules observed transitioning between clockwise and counter-clockwise circumferential movement, may correspond to protein complexes initiating the synthesis of a new glycan strand in the opposite direction.

The discovery of FtsZ treadmilling behaviour prompted the question of the role of this process in bacterial cell division^2^. One of the earlier hypotheses was that FtsZ treadmilling is required to distribute PG synthases around the division site homogeneously. This hypothesis was reinforced when direct evidence of the dependency of PG synthase movement on FtsZ treadmilling was provided for *E. coli*^4^ and *B. subtilis*^3^. In *E. coli*, FtsZ GTPase mutants reduced the population size of fast-moving (inactive) FtsWI complexes and, therefore, the overall velocity of directionally moving PG synthases^4,30^. In *B. subtilis*, a reduction of FtsZ treadmilling speed caused directionally moving PG synthases to slow down or become immobile^3^. However, we did not observe a similar behaviour in *S. aureus*. In our experiments, when FtsZ treadmilling was impaired either by introducing the GTPase mutation T111A into FtsZ or by treating cells with PC190723, the velocities of directionally moving FtsW and DivIB remained unchanged. In *S. aureus*, cytokinesis is biphasic and FtsZ treadmilling is only essential during the initial phase (before arrival of MurJ), but becomes dispensable for septum constriction afterwards^14^. We initially thought that maybe a dependency of FtsW or DivIB directional movement on FtsZ treadmilling could only be observed during that initial phase. However, that was not the case, as no such dependency was observed at any stage of cytokinesis (Supplementary Fig. 10). This result is in contrast with the studies of *E. coli*^4^ and initial studies in *B. subtilis*^3^, but in line with a previous report on *S. pneumoniae*, in which septal PG synthase movement depends on PG precursor availability, rather than FtsZ treadmilling^31^. Interestingly, recent data in *B. subtilis* suggests that the movement of PBP2B (orthologue of *S. aureus* PBP1) is not associated with FtsZ treadmilling also in this organism^46^. Although our data suggest that the directional movement of FtsW is uncoupled from FtsZ treadmilling, we cannot exclude that FtsW molecules may track with FtsZ filaments for a very short time, while spending most of the time actively synthesizing PG.

The question therefore remains regarding the essential function of FtsZ treadmilling in cell division*. E. coli* mutants with severely reduced FtsZ treadmilling were substantially altered in the ultrastructure of the septal cell wall and showed polar morphology defects^4^. In *B. subtilis*, FtsZ treadmilling is essential for Z-ring condensation, guides the initiation of constriction and is rate limiting for cell division^3,5^. We now show that *S. aureus* cells impaired in FtsZ treadmilling have constriction defects and altered morphology. However, contrarily to *B. subtilis*, the rate of septum synthesis remained unchanged even in the presence of PC190723, which completely stops FtsZ treadmilling^14^. Overall, we propose that, in *S. aureus*, FtsZ treadmilling does not determine the rate of septal PG synthesis, but rather constitutes a mechanism for ensuring uniformity of septum structure and hence the formation of two equally sized daughter cells.

We addressed one final question, regarding the existence of one or two subpopulations of processively moving PG synthases in *S. aureus*. In Gram-negative *E. coli*, directionally moving FtsWI exists in two motile populations: actively PG-synthesizing complexes move slowly (~8 nm s^−1^) relative to their enzymatically inactive counterparts (~30 nm s^−1^), and only the latter scale with FtsZ treadmilling speed (~28 nm s^−1^)^4,30^. Two motile populations of FtsW were also observed in Gram-negative *C. crescentus*^36^. We provide three pieces of evidence to support the conclusion that, in *S. aureus*, FtsW exists in a single motile population that depends on PG synthesis and not on FtsZ treadmilling: (1) FtsW moved with a slower velocity than treadmilling FtsZ filaments, (2) FtsW velocity remained unchanged when FtsZ treadmilling was impaired and (3) inhibition of PG synthesis slowed down or completely stopped FtsW, but not FtsZ treadmilling, and did not reveal a fast (inactive) FtsW subpopulation. Importantly, data obtained concurrently with ours in *B. subtilis* show that septal PG synthases in this organism also exist in a single motile population that is not dependent on FtsZ treadmilling for directional movement^46^. Given that two populations of directionally moving FtsW were detected only in Gram-negative species so far, it is tempting to speculate that FtsW dynamics may correlate with the different amounts of PG produced by bacteria. Gram-positive PG contains many layers and is 30–100 nm thick, whereas Gram-negative PG has only one to a few layers^47^. Thus, directionally moving FtsW/bPBP in Gram-positive bacteria could exist mostly in its active form, to synthesize the large amount of septal PG required for cell division. To produce a relatively small amount of septal PG at a similar synthesis rate, FtsW/bPBP in Gram-negative species may spend more time in its enzymatically inactive state, during which it would track with treadmilling FtsZ filaments.

## Methods

### Bacterial growth conditions

Strains and plasmids used in this study are listed in Supplementary Tables 4 and 5. *E. coli* strains were grown on Luria–Bertani agar (VWR) or in Luria–Bertani broth (VWR) with aeration at 37 °C. *S. aureus* strains were grown on tryptic soy agar (VWR), in TSB (Difco) or in M9 minimal medium (KH_2_PO_4_ 3.4 g l^−1^, VWR; K_2_HPO_4_ 2.9 g l^−1^, VWR; di-ammonium citrate 0.7 g l^−1^, Sigma-Aldrich; sodium acetate 0.26 g l^−1^, Merck; glucose 1% (w/v), Merck; MgSO_4_ 0.7 mg l^−1^, Sigma-Aldrich; CaCl_2_ 7 mg l^−1^, Sigma-Aldrich; casamino acids 1% (w/v), Difco; minimum essentrial medium amino acids 1×, Thermo Fisher Scientific; and minimum essential medium vitamins 1×, Thermo Fisher Scientific) at 200 rpm with aeration at 37 °C, 30 °C or 25 °C. When necessary, culture media were supplemented with antibiotics (100 μg ml^−1^ ampicillin, Sigma-Aldrich; 10 μg ml^−1^ erythromycin, Apollo Scientific; and 10 μg ml^−1^ chloramphenicol, Sigma-Aldrich). Unless otherwise specified, 5-bromo-4-chloro-3-indolyl β-d-galactopyranoside (X-Gal, Apollo Scientific) was used at 100 μg ml^−1^, IPTG (Apollo Scientific) was used at 0.5 mM and Atc (Sigma-Aldrich) was used at 2 ng ml^−1^.

### Construction of *S. aureus* strains

Oligonucleotides used in this study are listed in Supplementary Table 6. Cloning of FtsZ(T111A) mutants and tagged protein fusions in *S. aureus* was done using the following general strategy: plasmids were propagated in *E. coli* strain DC10B and purified from overnight cultures supplemented with the relevant antibiotics. Plasmids were then introduced into electrocompetent *S. aureus* RN4220 cells as previously described^48^ and transduced to JE2 or COL using phage 80α (ref. ^49^). Antibiotic marker-free allelic replacements in the *S. aureus* chromosome were performed using plasmids that allow for double homologous recombination events at selected genome sites. Constructs were confirmed by polymerase chain reaction (PCR) and by sequencing.

FtsZ(T111A) mutant strains were constructed using the pIMAY-Z vector^50^. An *ftsZ* allele encoding the GTPase point mutation T111A (by substitution of ACT for GCA at bases 331–333) was generated by amplifying a 989-bp upstream and a 898-bp downstream region of codon 111 from the JE2 genome using the primers 6703/6726 and 6727/6700, respectively. The two fragments were joined by overlap PCR using the primers 6703/6700, digested with SmaI/SalI and cloned into SmaI/XhoI-digested pIMAY-Z. The resulting plasmid pIMAY-Z-ftsZ(T111A) was electroporated into RN4220 and transduced into JE2, JE2 EzrA–sGFP and COL EzrA–sGFP derivative strains. Integration excision by a double homologous recombination event was performed at 30 °C; the *ftsZ* genomic region was sequenced to confirm the presence of the T111A mutation, and the chromosomal DNA of the JE2 EzrA–sGFP FtsZ(T111A) strain was sent for whole-genome sequencing.

Strains producing FtsW-HT, MurJ-HT, PBP4-HT or GpsB-HT fusions from their respective native genomic locus were constructed by allelic replacement strategies using the pMAD vector^51^. In brief, DNA fragments with approximately 800 bp spanning 3′ ends (excluding stop codons) of the *ftsW*, *murJ*, *pbp4* and *gpsB* genes from JE2 were amplified using the primers 7373/7142, 7376/6785, 7370/6783 and 7766/7597, respectively. A codon-optimized *ht* sequence was synthesized (Integrated DNA Technologies; Supplementary Information) and amplified with the primer pairs 7031/7369 (for *ftsW-ht*), 6715/7369 (for *murJ-ht* and *pbp4-ht*) or 6715/7769 (for *gpsB-ht*). Corresponding DNA fragments were joined either by overlap PCR using the primers 7373/7369 (to generate *ftsW-ht*) or by SalI digestion followed by ligation (to generate *murJ-ht, pbp4-ht* and *gpsB-ht*). The downstream region of the *ftsW*, *murJ*, *pbp4* and *gpsB* genes from JE2 were amplified using the primers 7374/7375, 7377/7378, 7371/7372 and 7770/7771, respectively. Corresponding DNA fragments were joined either by KpnI digestion followed by ligation (for *ftsW-ht*, *murJ-ht* and *pbp4-ht*) or by overlap PCR using the primers 7766/7771 (for *gpsB-ht*). The full constructs were then digested with EcoRI/BamHI (for *ftsW-ht, murJ-ht* and *pbp4-ht*) or SmaI/BamHI (for *gpsB-ht*) and cloned into equally digested pMAD. Integration and excision of the pMAD derivatives in JE2 EzrA–sGFP and COL EzrA–sGFP derivative strains by a double homologous recombination event that led to allelic exchange was performed as previously described^51^.

Strains ectopically producing HT-DivIB, RodA-HT, FtsW-HT, FtsZ-HT, EzrA-HT and iST-PBP1 were constructed using pBCB13 and pBCB43 vectors^52,53^, which are derivatives of pMAD that allow gene expression from the ectopic *spa* locus under the control of the (IPTG-inducible) *spac* promoter and the (Atc-inducible) *xyl-tetO* promoter, respectively. Briefly, a codon-optimized *ht* sequence (Supplementary Information) was amplified with the primer pairs 6713/6714 and 6715/6716, digested with SmaI and cloned into equally digested pBCB13, to generate plasmids pBCB13-Nht and pBCB13-htC, respectively. The in-frame insertion of a coding sequence into the multiple cloning site of pBCB13-Nht or pBCB13-htC results in a translational fusion containing the HT sequence either at the gene product’s N- or C-terminus, respectively. The *divIB* full-length and truncated coding sequences (each excluding the start codon) were amplified from JE2 using primers 7590/7591 and 7590/9171, digested with XhoI/EagI and ligated into SalI/EagI-digested pBCB13-Nht, to generate pBCB13-htdivIB and pBCB13-htdivIB(Δγ), respectively. The *rodA* full-length coding sequence (excluding the stop codon) was amplified from JE2 using the primers 7594/7595, digested with EagI/SalI and ligated into equally digested pBCB13-htC, to generate pBCB13-rodA-ht. For the construction of pBCB43 derivatives, the *ftsW, ftsZ* and *ezrA* full-length coding sequences (excluding the stop codons) were amplified from JE2 using the primers 9247/7142, 7191/7267 and 7139/7140, respectively. Active-site mutant derivatives of *ftsW* were amplified from plasmids pCNX-ftsW(W121A)sgfp and pCNX-ftsW(D287A)sgfp^18^ using the same *ftsW* primers. Note that primer 9247 contains a *tetO* sequence for an improved TetR repression. A codon-optimized *ht* sequence was amplified with the primer pair 7031/7034 and joined with the *ftsW, ftsZ* and *ezrA* coding sequences by overlap PCR using the primer pairs 9247/7034, 7191/7034 and 7139/7034, respectively. The full constructs were then SmaI/EagI-digested and cloned into equally digested pBCB43. To generate pBCB43-ist-pbp1, the *pbp1* full-length coding sequence (excluding the start codon) was amplified from JE2 using the primer pair 3810/9647 and the *st* coding sequence amplified from pST (T7)-2 (New England Biolabs; Supplementary Information) using the primer pair 9631/9632. Note that primer 9631 adds an *i-tag* sequence^54^ (Supplementary Information) for elevated expression levels in *S. aureus*. The two DNA fragments were joined by overlap PCR using the primer pair 9631/9647, digested with SmaI/XbaI and ligated into SmaI/NheI-digested pBCB43. Integration and excision of the pBCB13 and pBCB43 derivatives in JE2 EzrA–sGFP and COL EzrA–sGFP derivative strains by a double homologous recombination event that led to gene replacement at the *spa* locus was performed as previously described^52^.

### Growth curves of *S. aureus* strains

To assess growth of JE2 EzrA–sGFP derivative strains encoding ST and HT protein fusions, overnight cultures in TSB were back-diluted 1:1,000 into fresh media. A 200 µl sample of each culture was added to a well in a 96-well plate. Plates were incubated shaking at 37 °C and the optical density at 600 nm (OD_600_) was recorded every 15 min for 8 h in a 96-well plate reader (Biotek Synergy Neo2). Cells producing HT-DivIB, EzrA-HT and iST-PBP1 were grown in the presence of 0.5 mM IPTG, 0.5 ng ml^−1^ Atc and 2 ng ml^−1^ Atc, respectively.

To determine the cell growth rates of JE2 EzrA–sGFP, COL EzrA–sGFP and ColPBP1TP EzrA–sGFP derivative strains in fast and slow growth conditions, overnight cultures in TSB at 37 °C, 30 °C or 25 °C were back-diluted to an OD_600_ of 0.01 in 50 ml TSB rich medium or M9 minimal medium, in 250 ml Erlenmeyer flasks. Cells were grown with shaking at 200 rpm at 37 °C, 30 °C or 25 °C in triplicate. A total of 1 ml samples were taken every 20 min, 30 min or 40 min to record the OD_600_ using a spectrophotometer (Biochrom Ultrospec 2100 Pro). Growth rates were calculated for each strain during its exponential growth phase.

### Protein in-gel fluorescence detection and western blot analysis

To assess the integrity of ST and HT protein fusions, JE2 EzrA–sGFP derivative strains were grown to mid-exponential phase (OD_600_ of 0.6–0.8) in 50 ml TSB at 37 °C. Cells producing ectopic HT-DivIB, EzrA-HT and iST-PBP1 were grown in the presence of 0.5 mM IPTG, 0.5 ng ml^−1^ Atc and 2 ng ml^−1^ Atc, respectively. Cells producing FtsW-HT wild-type and mutant derivatives from the ectopic *spa* locus were grown in the presence of 0.2 ng ml^−1^ Atc. Cells were harvested by centrifugation, re-suspended in 0.3 ml fresh TSB and labelled with 500 nM of either JF549-HTL (red fluorescent Janelia Fluor 549 HT ligand) or JF549-cpSTL (red fluorescent Janelia Fluor 549 cell-permeable ST ligand) for 20 min at 37 °C. Cells were cooled on ice, washed one time with 1 ml phosphate-buffered saline (PBS) and re-suspended in 0.3 ml PBS supplemented with complete mini protease Inhibitor Cocktail (Roche). Cell suspensions were transferred to lysis tubes containing glass beads and subjected to mechanical disruption in a homogenizer SpeedMill Plus (Bioanalytik Jena) programmed to six 1 min cycles. Glass beads and cell debris were removed in two steps of centrifugation each for 1 min at 3,400*g*. A total of 25 µl of non-boiled protein sample were loaded on 12% Mini-Protean TGX pre-cast gels (Bio-Rad) and the proteins separated by sodium dodecyl-sulfate polyacrylamide gel electrophoresis. Gels were imaged in a FujiFilm FLA-5100 imaging system. EzrA–sGFP fluorescence was detected using 473 nm laser/Cy3 filter, JF549 fluorescence was detected using 532 nm laser/LPB filter and pre-stained molecular weight marker (PageRuler, Thermo Scientific) was detected using 635 nm laser/LPFR filter settings. Gels were post-stained with InstantBlue Coomassie stain (Abcam).

To detect FtsZ wild-type protein and its T111A mutant variant, JE2 EzrA–sGFP FtsW-HT and COL EzrA–sGFP FtsW-HT derivative strains were grown to mid-exponential phase (OD_600_ of 0.6–0.8) in 50 ml TSB at 30 °C. Cells were cooled on ice, harvested by centrifugation and re-suspended in 0.3 ml PBS supplemented with complete mini protease Inhibitor Cocktail (Roche). Whole cell protein extracts were obtained as described above and protein concentrations determined using Bradford reagent (Thermo Scientific). A total of 2.5 μg of total protein were loaded on 12% Mini-Protean TGX pre-cast gels (Bio-Rad). Separated proteins were then transferred to a nitrocellulose membrane using a Trans-Blot Turbo RTA Mini 0.2 μm Nitrocellulose Transfer Kit and Trans-Blot Turbo system (Bio-Rad). The membrane was cut to separate the regions above and below approximately 70 kDa. The top part of the membrane was incubated with Sypro-Ruby stain (Invitrogen) following the manufacturer’s instructions to label high-molecular weight proteins. The bottom part of the membrane containing FtsZ was blocked with 5% milk, followed by consecutive incubations with an anti-FtsZ antibody (1:2,000 dilution) for 16 h at 4 °C and with a secondary fluorescent antibody (Alexa-488 anti-sheep diluted 1:50,000; Thermo Fisher) for 1 h at room temperature. Alexa-488 and Sypro-Ruby fluorescence detection was performed in an iBright Imaging System (Invitrogen).

To detect the PBP1 wild-type protein in the JE2 strain grown in various conditions, the same procedure as described above was followed, except that an anti-PBP1 antibody (1:1,000 dilution) and a secondary horseradish peroxidase antibody (anti-rabbit diluted 1:50,000; GE Healthcare) combined with an Amersham ECL Prime Western Blotting Detection Reagent (GE Healthcare) were used.

Uncropped images of gels and blotted membranes can be found in Supplementary Figs. 14 and 15.

### Transmission electron microscopy

Transmission electron microscopy was essentially performed as previously described^55^. Briefly, cells of strains JE2 and JE2 FtsZ(T111A) were grown in TSB rich medium at 37 °C to mid-exponential phase (OD_600_ of 0.6–0.8) and harvested by centrifugation. Cell pellets were re-suspended and fixed in wash buffer (0.1 M PIPES, pH 7.2) containing 2.5% glutaraldehyde (Carl Roth) and incubated on ice for 1 h, followed by three wash steps with wash buffer. Cells were then post-fixed in wash buffer containing 1% osmium tetroxide (Acros Organics) for 1 h at 4 °C and washed five times with MilliQ H_2_O. Cells were embedded in 2% low melting point agarose, stained with 0.5% uranyl acetate (Analar) in H_2_O overnight at 4 °C and washed twice with MilliQ H_2_O. Dehydration was performed at 4 °C by increasing the ethanol concentration in samples gradually from 30% to 50%, 70%, 80%, 90% and 100% for 10 min in each step. The final step was repeated one time and ethanol exchanged with ice-cold acetone in two steps for 10 min and 20 min at room temperature. Infiltration and embedding were performed using Spurr’s resin (Science Services) and samples were polymerized for 24–48 h at 60 °C. Ultrathin sections (70 nm) were generated with an EM UC7 ultramicrotome (Leica), mounted on copper palladium slot grids coated with 1% formvar (Agar Scientific) in chloroform and post-stained with uranyl acetate and Reynold’s lead citrate for 5 min each. Transmission electron microscopy imaging was performed at 120 kV using a FEI Tecnai G2 Spirit BioTWIN microscope equipped with an Olympus-SIS Veleta CCD Camera.

### Fluorescence microscopy

To study the localization of ST and HT protein fusions, JE2 EzrA–sGFP, COL EzrA–sGFP and ColPBP1TP EzrA–sGFP derivative strains were grown overnight in TSB and diluted 1:200 in fresh TSB followed by incubation with shaking at 37 °C. For cells producing HT-DivIB, EzrA-HT and iST-PBP1 the medium was supplemented with 0.5 mM IPTG, 0.5 ng ml^−1^ Atc and 2 ng ml^−1^ Atc, respectively. Cells producing FtsZ-HT and RodA-HT were grown in the absence of inducer, as leaky expression from the *xyl-tetO* and *spac* promoters, respectively, was sufficient for imaging. After cells reached mid-exponential growth phase (OD_600_ of 0.6–0.8), 500 nM of either JF549-HTL or JF549-cpSTL were added to 1 ml of culture followed by incubation with shaking for 20 min at 37 °C. Where indicated, cells were simultaneously treated with 2 µg ml^−1^ vancomycin (Sigma-Aldrich) for 20 min at 37 °C. Cells were pelleted by centrifugation for 1 min at 9,300*g*, washed one time, re-suspended in PBS and spotted on a microscope slide covered with a thin layer of 1.5% TopVision agarose (Thermo Fisher) in PBS. Images were acquired with a Zeiss Axio Observer microscope equipped with a Plan-Apochromat 100×/1.4 oil Ph3 objective, a Retiga R1 CCD camera (QImaging), a white-light source HXP 120 V (Zeiss) and the software ZEN blue v2.0.0.0 (Zeiss). For image acquisition, the filters (Semrock) Brightline GFP-3035B (sGFP) and Brightline TXRED-4040B (JF549) were used.

To analyse the cell size and cell cycle of *S. aureus*, overnight cultures in TSB of strains COL EzrA–sGFP, COL EzrA–sGFP FtsZ(T111A) and ColPBP1TP EzrA–sGFP were inoculated in quadruplicates from independent single colonies and incubated shaking at 30 °C or 37 °C. Cultures were diluted 1:200 into fresh TSB followed by incubation with shaking at the same temperatures. A total of 1 ml of mid-exponential growth phase cells (OD_600_ of 0.6–0.8) was mixed with 5 µg ml^−1^ Nile red (Invitrogen) and 1 µg ml^−1^ Hoechst 33342 (Invitrogen) and incubated shaking for 5 min. Cells were washed one time with PBS and spotted on a pad of 1.5% TopVision agarose (Thermo Fisher) in PBS, and mounted in a Gene Frame (Thermo Fisher) on a microscope slide. Cells were then imaged by structured illumination microscopy (SIM) using an Elyra PS.1 microscope (Zeiss) equipped with a Plan-Apochromat 63×/1.4 oil differential interference contrast M27 objective. SIM images were acquired using five grid rotations with 34 µm grating period for the 561 nm laser (100 mW, at 50% maximal power) and 23 µm grating period for the 405 nm laser (100 mW, at 100% maximal power). Images were captured using a PCO Edge 5.5 camera and reconstructed using the software ZEN black v8.1.0.484. Following SIM image reconstruction, cell size was measured and cells classified according to their cell cycle phase (phase 1: before initiation of membrane constriction at mid cell; phase 2: ongoing membrane constriction for septum synthesis; and phase 3: closed septum), using the software eHooke^56^.

To evaluate localization of PG synthesis activity, strains JE2 EzrA–sGFP FtsW-HT and COL EzrA–sGFP FtsW-HT producing wild-type FtsZ or FtsZ(T111A) were grown to mid-exponential growth phase (OD_600_ of 0.6–0.8) in TSB rich medium at 30 °C and then dually labelled with 500 nM JF549-HTL for 20 min and with 25 µM fluorescent d-amino acid HADA for 10 min. Cells were washed one time with PBS and spotted on a pad of 1.5% TopVision agarose (Thermo Fisher) in PBS, and mounted in a Gene Frame (Thermo Fisher) on a microscope slide. Imaging was performed in a DeltaVision OMX SR microscope equipped with an Olympus 60X PlanApo N/1.42 oil differential interference contrast (DIC) objective and two PCO Edge 5.5 sCMOS cameras (one for differential interference contrast, HADA and sGFP; one for JF549 and JFX650). The software AcquireSR v4.4 (GE Healthcare) was programmed to acquire *Z*-stacks of five images with a step size of 300 nm using a 488 nm laser (100 mW, at 20% maximal power), a 568 nm laser (100 mW, at 20% maximal power) and a 405 nm laser (100 mW, at 30% maximal power), each with an exposure time of 100 ms. The software SoftWorRx (v7.2.1) was used for maximum intensity projection (MIP) of five images from each *Z*-stack, fluorescence channel alignment and image deconvolution.

To determine EzrA–sGFP ring constriction rates, JE2 EzrA–sGFP, COL EzrA–sGFP and ColPBP1TP EzrA–sGFP derivative strains were grown in duplicate overnight in TSB and diluted 1:200 in fresh TSB followed by incubation with shaking at 37 °C or 30 °C. Exponentially growing cells (OD_600_ of 0.6–0.8) were harvested by centrifugation for 1 min at 9,300*g*, re-suspended in 30 µl fresh TSB and spotted on a microscope slide covered with a thin layer of 1.5% TopVision agarose (Thermo Fisher) in TSB:PBS (1:1). Where indicated, 8 µg ml^−1^ DMPI or 5 µg ml^−1^ PC190723 were added to 1 ml of culture for 2 min at 37 °C before cell harvest and cells were maintained in the presence of antibiotic during washing and imaging. The time between the cells contacting the pad and the start of image acquisition was 5 min. Images were acquired in a DeltaVision OMX SR microscope (GE Healthcare) in SIM mode. SIM images (three phase shifts and three grid rotations) were acquired every 3 min for 75 min (90 min for imaging at 30 °C for COL background, 60 min for JE2 background) using a 488 nm laser (100 mW, at 10% maximal power and 5% for JE2 background) with an exposure time of 25 ms (50 ms for JE2 background). Images were reconstructed using SoftWoRx v7.2.1 and aligned using NanoJ v2.1RC1 drift correction^57^. One-pixel straight lines were drawn on constricting EzrA–sGFP rings to generate space–time kymographs in ImageJ/Fiji v1.53^58^. Only cells visibly constricting over a minimum of five consecutive time frames and that completed septum closure during a recorded time series were used for analysis, except for DMPI samples where septum closure events were seldom.

To analyse EzrA–sGFP movement dynamics (used as a proxy for FtsZ treadmilling), JE2 EzrA–sGFP, COL EzrA–sGFP and ColPBP1TP EzrA–sGFP derivative strains were grown in duplicate overnight in TSB and diluted 1:200 in fresh TSB followed by incubation with shaking at 37 °C or 30 °C. Exponentially growing cells (OD_600_ of 0.6–0.8) were harvested by centrifugation for 1 min at 9,300*g*, re-suspended in 30 µl fresh TSB, and spotted on a pad of 1.5% molecular biology grade agarose (Bio-Rad) in M9 minimal medium mounted in a Gene Frame (Thermo Fisher) on a microscope slide. Where indicated, cells were treated with DMPI and PC190723 as described above. The time between the cells contacting the pad and the start of image acquisition was 5 min and image acquisition was done during a maximum period of 30 min. Imaging was performed in a DeltaVision OMX SR microscope equipped with a hardware-based focus stability (HW UltimateFocus) and an environmental control module set to 37 °C or 30 °C. *Z*-stacks of three images with a step size of 500 nm were acquired every 3 s for 3 min using a 488 nm laser (100 mW, at 10% maximal power) with an exposure time of 50 ms. MIP of three images from each *Z*-stack and subsequent image deconvolution was performed for each time frame in SoftWoRx v7.2.1. All 61 time frames were aligned using NanoJ v2.1RC1 drift correction^57^ and then used to perform MIP for the drawing of one-pixel freehand lines over EzrA–sGFP in late pre-divisional cells, in which nascent *Z*-rings appear sparse and D-shaped. Space–time kymographs were then generated by extracting fluorescence intensities along drawn lines from individual time frames using the software ImageJ/Fiji v1.53 (ref. ^58^). FtsZ treadmilling speed was calculated in nm s^−1^ by measuring the slopes of straight lines drawn on diagonals spanning the entire width in kymographs and corresponding to circumferentially moving EzrA–sGFP.

To perform single-molecule imaging, JE2 EzrA–sGFP, COL EzrA–sGFP and ColPBP1TP EzrA–sGFP derivative strains producing ST or HT protein fusions were grown in triplicate overnight in TSB and diluted 1:200 in fresh TSB rich medium or M9 minimal medium. For cells producing HT-DivIB, EzrA-HT and iST-PBP1, the medium was supplemented with 0.5 mM IPTG, 0.5 ng ml^−1^ Atc and 2 ng ml^−1^ Atc, respectively. Cells were grown with shaking at 37 °C, 30 °C or 25 °C to mid-exponential growth phase (OD_600_ of 0.4–0.8). A total of 1 ml of cell culture was then mixed either with 5 nM JFX650-STL (far-red fluorescent Janelia Fluor X 650 ST ligand) or with JF549-HTL at concentrations ranging from 10 pM to 250 pM (Supplementary Tables 2 and 3) and incubated with shaking for 20 min at 37 °C, 30 °C or 25 °C. Cells were harvested by centrifugation for 1 min at 9,300*g*, re-suspended in 30 µl fresh M9 minimal medium, spotted on a pad of 1.5% molecular biology grade agarose (Bio-Rad) in M9 minimal medium, mounted in a Gene Frame (Thermo Fisher) on a microscope slide and covered with a glass coverslip pre-washed with changes of ethanol, acetone, 0.1 M KOH and MilliQ H_2_O. Where indicated, cells were treated with DMPI or PC190723 as described above, or with 10 µg ml^−1^ imipenem (Apollo Scientific) or 2 µg ml^−1^ vancomycin (Sigma-Aldrich), for 20 min at 37 °C before harvest and cells maintained in the presence of antibiotic during washing and imaging. The time between the cells contacting the pad and the start of image acquisition was 5 min and image acquisition was done during a maximum period of 30 min. Imaging was performed in a DeltaVision OMX SR microscope equipped with a hardware-based focus stability (HW UltimateFocus) and an environmental control module set to 37 °C, 30 °C or 25 °C. *Z*-stacks of three epifluorescence images with a step size of 500 nm were acquired every 3 s (or every second for a frame rate of 1 Hz) for 3 min (or for 2 min) using a 568 nm laser (100 mW, at 10% maximal power; for JF549-labelled HT protein fusions) or a 640-nm laser (100 mW, at 30% maximal power; for JFX650-labelled iST-PBP1), each with an exposure time of 800 ms (or 300 ms for 1 Hz image acquisitions). Additional *Z*-stacks were acquired in the first and the last time frames of every time series to record EzrA–sGFP fluorescence using a 488 nm laser (100 mW, at 10% maximal power) with an exposure time of 100 ms and to image cells in brightfield. MIP of three images from each *Z*-stack acquired in both fluorescence channels and fluorescence channel alignment was performed for each time frame using SoftWoRx v7.2.1. One-pixel freehand lines were drawn on the EzrA–sGFP signal in the last time frame and space–time kymographs were generated using ImageJ/Fiji v1.53^58^ by extracting fluorescence intensities from recorded single molecules in all 61 time frames.

### Single-molecule tracking data analysis

Spots corresponding to fluorescence signal from single molecules of labelled HT and ST protein fusions were detected in TrackMate v.7.2.0 (ref. ^59^) using the Laplacian of Gaussian filter with subpixel localization, a blob diameter of 400 nm and a quality threshold of 3. Tracks were generated by linking spots detected in two consecutive time frames (3 s or 1 s interval) using the simple linear assignment problem tracker with a maximum linkage distance of 125 nm and no frame gaps allowed. Obtained tracks were filtered for a minimum number of spots of 30 for 0.33 Hz image acquisitions (equivalent to a duration of ≥87 s) or of 60 for 1 Hz image acquisitions (equivalent to a duration of ≥59 s). To calculate the percentage of cells containing a track, cells imaged in brightfield were counted using Otsu thresholding in ImageJ/Fiji v1.53 (ref. ^58^). All further analysis was done by exporting the subpixel coordinates for each spot in a track from TrackMate v.7.2.0 to be used in an in-house developed Python tool^60,61^.

To calculate single-molecule motion in all three spatial dimensions of each detected molecule, an ellipse was manually drawn on the EzrA–sGFP signal for all cells with a directionally moving FtsW, PBP1 or DivIB molecule. The angle of the division ring relative to the image acquisition plane ($\Theta$) was determined for all drawn ellipses by calculating the arccosine of the ratio between the minor axis, $m$, and major axis, $M$ (equation (1)). Each track was then approximated to a track overlaying the division ring, by computing, for each track point, the closest point on the ellipse and projecting it onto a three-dimensional ring (Fig. 3a and Supplementary Video 6).

1 $$\Theta =\arccos \left(\frac{m}{M}\right).$$

For each trajectory, $(x\left(t\right),y\left(t\right))$ of length $L$, the mean squared displacement (MSD) is calculated as a time average of the MSDs in each dimension as^62^

2 $${\mathrm{MSD}}_{x}\left(t\right)=\frac{1}{L-t}\mathop{\sum }\limits_{i=1}^{L-t}{\left[x\left({t}_{i}+t\right)-x\left({t}_{i}\right)\right]}^{2},$$

3 $${\mathrm{MSD}}_{y}\left(t\right)=\frac{1}{L-t}\mathop{\sum }\limits_{i=1}^{L-t}{\left[\,y\left({t}_{i}+t\right)-y\left({t}_{i}\right)\right]}^{2},$$

4 $$\mathrm{MSD}\left(t\right)={\mathrm{MSD}}_{x}+{\mathrm{MSD}}_{y}.$$

It is known that for pure Brownian motion the MSD is linear with time while for anomalous diffusion it follows a power law scaling. Taking $D$ as a constant that depends on the diffusion coefficient and other constraints, and α is the anomalous diffusion exponent^63^:

5 $${MSD}\left(t\right)=D.{t}^{\alpha }$$

To calculate *α*, the following linear regression was performed on the first 20 MSD points:

6 $$\log \left({MSD}\right)\left(t\right)=\log \left(D\right)+{\rm{a}}.\log ({\rm{t}})$$

To determine single-molecule velocities, the approximated tracks were unwrapped and sectioned by testing a maximum of three possible breakpoints (maximum of four sections). Possible sections and breakpoints were tested by performing linear regressions between every putative breakpoint, ensuring that all sections have a minimum length of four spots. The best possible sections, using zero to three breakpoints, were selected by calculating the mean square error equation (7) and choosing the combination of sections that has the minimum average MSE of all sections, weighted by section length. Velocities were calculated as the slope of the linear regression of each section. Unless otherwise specified, velocity is given as the average of velocities (maximum of four) determined for each section of a track.

7 $$\mathrm{MSE}=\frac{1}{N}\sum {\left(X-{X}_{\mathrm{predicted}}\right)}^{2}$$

### *S. aureus* core divisome structure prediction and visualization

Predictions were generated for full-length *S. aureus* protein sequences using AlphaFold-Multimer^64^ and AlphaFold2 (ref. ^65^) as implemented in ColabFold^66^. All models for both methods produced similar predictions for the interface with PASTA domains (Cα root mean square deviation 0.12–0.55 Å between PASTA domains in the top-ranked AlphaFold-Multimer model and the nine other models after alignment to DivIB residues 263–398). The top-ranked AlphaFold-multimer model is shown in Supplementary Fig. 13.

### Statistics and reproducibility

Cell constriction rates were compared using a two-tailed Mann–Whitney *U* test in the software GraphPad Prism v9.1.0. All sample sizes and number of experimental replicates can be found in figures, figure legends and Supplementary Tables 1–3. No statistical method was used to pre-determine sample size, the experiments were not randomized, and the investigators were not blinded to allocation during experiments and outcome assessment. Data distributions were assumed to be normal, but this was not formally tested.

### Reporting summary

Further information on research design is available in the Nature Portfolio Reporting Summary linked to this article.

## Supplementary information

Supplementary Information Supplementary Tables 1–6, coding sequences, Figs. 1–15 and References.

Reporting Summary

Supplementary Video 1 FtsZ treadmilling in FtsZ(T111A) mutant and parental strains producing EzrA–sGFP and grown at 30 °C.

Supplementary Video 2 Septum constriction in FtsZ(T111A) mutant and parental strains producing EzrA–sGFP and grown at 30 °C.

Supplementary Video 3 FtsZ treadmilling in the strains COL EzrA–sGFP and ColPBP1TP EzrA–sGFP grown at 37 °C and in the absence or presence of indicated antibiotics.

Supplementary Video 4 Septum constriction in the strains COL EzrA–sGFP and ColPBP1TP EzrA–sGFP grown at 37 °C and in the absence or presence of indicated antibiotics.

Supplementary Video 5 Single-molecule tracking of FtsW-HT and HT-DivIB in the background of the JE2 EzrA–sGFP strain grown at 37 °C and in the absence or presence of indicated antibiotics. Cells were sparsely labelled with HTL-JF549 to resolve single molecules. Single-molecule and EzrA–sGFP images are overlayed with tracks, where blue (0 s) to red (180 s) indicates trajectory time.

Supplementary Video 6 Animation illustrating the analysis of single-molecule tracking data for molecules moving directionally around the division site of coccoid cells placed on a microscope slide.

Supplementary Video 7 Single-molecule tracking of FtsW-HT and HT-DivIB in FtsZ(T111A) mutant and parental strains grown at 30 °C. Cells were sparsely labelled with HTL-JF549 to resolve single molecules. Single-molecule and EzrA–sGFP images are overlayed with tracks, where blue (0 s) to red (180 s) indicates trajectory time.

Supplementary Video 8 Single-molecule tracking of FtsW-HT and HT-DivIB in the background of the strains COL EzrA–GFP and ColPBP1TP EzrA–sGFP grown at 37 °C. Cells were sparsely labelled with HTL-JF549 to resolve single molecules. Single-molecule and EzrA–sGFP images are overlayed with tracks, where blue (0 s) to red (180 s) indicates trajectory time.

Supplementary Data Numerical Source data for Supplementary Figs. 2, 3, 5, 6, 8, 10 and 12.

## Source data

Source Data Fig. 1 Numerical source data for Fig. 1.

Source Data Fig. 3 Numerical source data for Fig. 3.

Source Data Fig. 4 Numerical source data for Fig. 4.

Source Data Fig. 5 Numerical source data for Fig. 5.

Source Data Fig. 6 Numerical source data for Fig. 6.

## Data Availability

A reporting summary for this article is available as a Supplementary Information file. Unprocessed image data for Supplementary Videos 1–5, 7 and 8 are available on Figshare at https://figshare.com/projects/SaureusDivisomeDynamics/191457. Source data are provided with this paper.

## Code availability

Custom software is available on the Pinho lab GitHub page at https://github.com/BacterialCellBiologyLab/AureusSpeedTracker.
