# Supplementary material for: Cell constriction requires processive septal peptidoglycan synthase movement independent of FtsZ treadmilling in Staphylococcus aureus
Source: Nat Microbiol. 2024 Mar 13;9(4):1049–63. doi: 10.1038/s41564-024-01629-6 (PMC10994846; doi:10.1038/s41564-024-01629-6)
Supplement: Supplementary file 1 — Supplementary Tables 1–6, coding sequences, Figs. 1–15 and References. [file 41564_2024_1629_MOESM1_ESM.pdf]

nature microbiology

Article https://doi.org/10.1038/s41564-024-01629-6

Cell constriction requires processive septal peptidoglycan synthase movement independent of FtsZ treadmilling in *Staphylococcus aureus*

In the format provided by the authors and unedited

Table of contents

- Supplementary Tables 1-6
- Coding sequences for protein tags
- Supplementary Figures 1-15
- References

Table S1. Overview of FtsZ treadmilling speeds, septum constriction rates, and single-molecule velocities determined in this study. Mean and standard deviation (SD) of FtsZ treadmilling speeds, EzrA-sGFP ring constriction rates, and the velocity of FtsW-HT and HT-DivIB single molecules. T, growth temperature. n.d., not determined.

		FtsZ treadmilling speed (nm/s)		Septum constriction rate (nm/min)		FtsW-HT velocity (nm/s)		HT-DivIB velocity (nm/s)	
T (°C)	Genetic background, antibiotic treatment	Mean±SD	*n*	Mean±SD	*n*	Mean±SD	*n*	Mean±SD	*n*
30	JE2	40.2±9.0	171	14.6±3.8	20	10.6±3.0	623	11.0±3.3	897
	JE2 FtsZ(T111A)	0.4±0.5	15	15.1±5.6	20	10.7±3.7	536	11.2±3.7	336
	COL	39.6±8.5	83	18.0±4.6	20	9.4±3.6	262	9.6±3.3	131
	COL FtsZ(T111A)	0.3±0.5	21	18.0±3.2	20	9.3±3.3	480	10.2±3.7	189
37	JE2	60.6±9.2	32	n.d.		15.7±4.3	486	16.5±5.0	194
	JE2, PC190723	0.4±0.5	9	n.d.		16.0±4.8	449	16.4±4.4	282
	COL	59.7±8.7	56	21.4±4.2	20	15.5±4.5	209	16.7±4.9	164
	COL, PC190723	0.3±0.5	6	22.5±3.0	20	14.0±4.4	230	15.3±4.6	144
	ColPBP1TP	61.8±11.7	51	9.3±1.9	20	8.8±4.8	321	9.2±3.9	191
	COL, DMPI	61.5±10.0	37	1.7±1.2	20	n.d.		n.d.	
	JE2, DMPI	n.d.		n.d.		4.9±2.6	227	6.4±3.0	343

Table S2. FtsW, PBP1 and DivIB move directionally along septal rings as opposed to other cell division proteins. Track statistics of protein fusions for various cell division and cell wall-related proteins studied by single-molecule imaging. Indicated strains were grown in TSB at 37°C and cells producing iST-PBP1, EzrA-HT or HT-DivIB derivatives from the ectopic *spa* locus were grown in the presence of 2 ng/ml Atc, 0.5 ng/ml Atc or 0.5 mM IPTG, respectively. Proteins fused to Halo-tag (HT) were labelled with the red fluorescent dye JF549-HTL at the indicated concentrations. iST-PBP1 was labelled with the far-red fluorescent dye JFX650-STL. Spot detection and tracking was performed in TrackMate (max. linkage distance, 125 nm; no frame gaps allowed). Obtained tracks were filtered for ≥30 spots per track (equivalent to a duration of ≥87 s). Only tracks overlapping with an EzrA-sGFP ring and an α_{MSD} ≥1 (track directionality) were included in this analysis. The number of tracks was normalized to the number of imaged cells (minimum 1194) for each sample. Θ indicates the angle between the imaging and cell division planes.

Strain	Dye conc. (pM)	Number of tracks	% of cells with track	Avg. α_{MSD}	Avg. track duration (s)	Avg. Θ (deg)
JE2 EzrA-sGFP	10	0	0.00	-	-	-
JE2 EzrA-sGFP FtsW-HT	10	125	5.13	1.48	120	51
JE2 EzrA-sGFP MurJ-HT	10	7	0.29	1.15	121	49
JE2 EzrA-sGFP PBP4-HT	10	0	0.00	-	-	-
JE2 EzrA-sGFP GpsB-HT	10	0	0.00	-	-	-
JE2 EzrA-sGFP spa-FtsZ-HT	10	0	0.00	-	-	-
JE2 EzrA-sGFP spa-EzrA-HT	10	0	0.00	-	-	-
JE2 EzrA-sGFP spa-HT-DivIB	10	22	1.23	1.51	132	53
JE2 EzrA-sGFP spa-HT-DivIB	50	63	3.12	1.51	112	53
JE2 EzrA-sGFP spa-HT-DivIB(Δγ)	50	1	0.03	1.22	105	31
JE2 EzrA-sGFP spa-RodA-HT	10	1	0.04	1.27	102	72
JE2 EzrA-sGFP spa-Pxyl-tetO	5,000	0	0.00	-	-	-
JE2 EzrA-sGFP spa-iST-PBP1	5,000	192	0.69	1.45	114	55

Table S3. Track statistics of FtsW and DivIB protein fusions studied by single-molecule imaging. JE2 EzrA-sGFP, COL EzrA-sGFP and ColPBP1TP EzrA-sGFP derivative strains were grown in the indicated conditions. Cells producing HT-DivIB or FtsW-HT derivatives from the ectopic *spa* locus were grown in the presence of 0.5 mM IPTG or 0.2 ng/ml Atc, respectively. HT-DivIB and FtsW-HT derivatives were labelled with the red fluorescent dye JF549-HTL. Tracking was performed in TrackMate (max. linkage distance, 125 nm; no frame gaps allowed). All tracks on top of EzrA-sGFP rings were filtered for number of spots in track (≥30, equivalent to ≥87 s) and α_{MSD} (≥1). The number of tracks was normalized to the number of imaged cells (minimum 3374) for each sample and tracks were obtained from at least three biological replicates. Θ indicates the angle between the imaging and cell division planes.

Fusion	Figure	Genetic background, growth temperature, growth medium/ antibiotic treatment/ protein variant	Dye conc. (pM)	Number of tracks	% of cells with track	Avg. α_{MSD}	Avg. track duration (s)	Avg. Θ (deg)
FtsW-HT	4a	JE2, 37°C, TSB	10	694	9.26	1.53	126	49
		JE2, 37°C, M9	50	472	7.13	1.48	131	64
		JE2, 30°C, TSB	10	685	9.91	1.43	141	56
		JE2, 25°C, TSB	10	385	4.14	1.36	148	46
	5a	JE2, 30°C	10	774	10.17	1.49	137	49
		JE2 FtsZ(T111A), 30°C	10	597	12.34	1.48	131	50
		COL, 30°C	20	344	4.65	1.41	137	54
		COL FtsZ(T111A), 30°C	20	530	6.29	1.42	134	50
	5c	JE2, 37°C	10	619	7.63	1.52	127	53
		JE2, 37°C, PC190723	10	560	6.41	1.51	126	53
		COL, 37°C	20	230	3.01	1.48	130	53
		COL, 37°C, PC190723	20	251	3.39	1.45	128	47
	6a	JE2, 37°C	10	619	7.63	1.52	127	53
		JE2, 37°C, imipenem	10	397	8.29	1.24	133	47
		JE2, 37°C, DMPI	10	243	4.46	1.27	135	54
		JE2, 37°C, vancomycin	10	5	0.10	1.19	105	24
	6c	COL, 37°C	20	230	3.01	1.48	130	53
		ColPBP1TP, 37°C	20	290	3.92	1.25	131	45
FtsW-HT(spa)	6e	JE2, 37°C, FtsW	200	250	3.76	1.52	121	57
		JE2, 37°C, FtsW(W121A)	200	59	0.38	1.37	116	48
		JE2, 37°C, FtsW(D287A)	200	121	0.90	1.32	111	53
HT-DivIB	4b	JE2, 37°C, TSB	50	499	5.72	1.51	120	52
		JE2, 37°C, M9	250	92	1.52	1.50	129	62
		JE2, 30°C, TSB	50	989	12.14	1.44	137	59
		JE2, 25°C, TSB	50	209	2.59	1.39	152	47
	5b	JE2, 30°C	50	1,073	12.10	1.50	131	48
		JE2 FtsZ(T111A), 30°C	50	386	8.20	1.52	127	51
		COL, 30°C	100	189	3.11	1.48	135	61
		COL FtsZ(T111A), 30°C	100	218	2.90	1.46	136	53
	5d	JE2, 37°C	50	250	5.89	1.50	120	56
		JE2, 37°C, PC190723	50	345	5.05	1.51	118	54
		COL, 37°C	100	217	2.30	1.50	124	58
		COL, 37°C, PC190723	100	181	1.97	1.48	125	56
	6b	JE2, 37°C	50	250	5.89	1.50	120	56
		JE2, 37°C, imipenem	50	386	7.77	1.32	133	45
		JE2, 37°C, DMPI	50	500	7.85	1.39	133	58
		JE2, 37°C, vancomycin	50	9	0.19	1.17	119	35
	6d	COL, 37°C	100	217	2.30	1.50	124	58
		ColPBP1TP, 37°C	100	178	2.24	1.32	132	45

Table S4. Bacterial strains used in this study.

Name	Description	Reference
Escherichia coli		
DC10B	Δ*dcm* in DH10B background; Dam methylation only; for cloning	1
Staphylococcus aureus		
RN4220	Restriction-negative derivative of NCTC8325-4	2
JE2	CA-MRSA strain	3
JE2 FtsZ(T111A)	JE2 *ftsZ::ftsZ*$_{T111A}$	This study
JE2 EzrA-sGFP	JE2 *ezrA::ezrA-sgfp*	4
JE2 EzrA-sGFP FtsZ(T111A)	JE2 *ezrA::ezrA-sgfp ftsZ::ftsZ*$_{T111A}$	This study
COL EzrA-sGFP	COL *ezrA::ezrA-sgfp;* derivative of HA-MRSA strain COL	4
COL EzrA-sGFP FtsZ(T111A)	COL *ezrA::ezrA-sgfp ftsZ::ftsZ*$_{T111A}$	This study
ColPBP1TP	COL *pbp1::pbp1*$_{S314A}$	5
ColPBP1TP EzrA-sGFP	COL *pbp1::pbp1*$_{S314A}$ *ezrA::ezrA-sgfp*	This study
JE2 EzrA-sGFP FtsW-HT	JE2 *ezrA::ezrA-sgfp ftsW::ftsW-halo*	This study
JE2 EzrA-sGFP FtsW-HT FtsZ(T111A)	JE2 *ezrA::ezrA-sgfp ftsW::ftsW-halo ftsZ::ftsZ*$_{T111A}$	This study
COL EzrA-sGFP FtsW-HT	COL *ezrA::ezrA-sgfp ftsW::ftsW-halo*	This study
COL EzrA-sGFP FtsW-HT FtsZ(T111A)	COL *ezrA::ezrA-sgfp ftsW::ftsW-halo ftsZ::ftsZ*$_{T111A}$	This study
ColPBP1TP EzrA-sGFP FtsW-HT	COL *pbp1::pbp1*$_{S314A}$ *ezrA::ezrA-sgfp ftsW::ftsW-halo*	This study
JE2 EzrA-sGFP spa-HT-DivIB	JE2 *ezrA::ezrA-sgfp* Δ*spa::*P$_{spac}$*-halo-divIB*	This study
JE2 EzrA-sGFP spa-HT-DivIB FtsZ(T111A)	JE2 *ezrA::ezrA-sgfp* Δ*spa::*P$_{spac}$*-halo-divIB ftsZ::ftsZ*$_{T111A}$	This study
COL EzrA-sGFP spa-HT-DivIB	COL *ezrA::ezrA-sgfp* Δ*spa::*P$_{spac}$*-halo-divIB*	This study
COL EzrA-sGFP spa-HT-DivIB FtsZ(T111A)	COL *ezrA::ezrA-sgfp* Δ*spa::*P$_{spac}$*-halo-divIB ftsZ::ftsZ*$_{T111A}$	This study
ColPBP1TP EzrA-sGFP spa-HT-DivIB	COL *pbp1::pbp1*$_{S314A}$ *ezrA::ezrA-sgfp* Δ*spa::*P$_{spac}$*-halo-divIB*	This study
JE2 EzrA-sGFP MurJ-HT	JE2 *ezrA::ezrA-sgfp murJ::murJ-halo*	This study
JE2 EzrA-sGFP PBP4-HT	JE2 *ezrA::ezrA-sgfp pbp4::pbp4-halo*	This study
JE2 EzrA-sGFP GpsB-HT	JE2 *ezrA::ezrA-sgfp gpsB::gpsB-halo*	This study
JE2 EzrA-sGFP spa-Pxyl-tetO	JE2 *ezrA::ezrA-sgfp* Δ*spa::*P$_{xyl\text{-}tetO}$	This study
JE2 EzrA-sGFP spa-FtsZ-HT	JE2 *ezrA::ezrA-sgfp* Δ*spa::*P$_{xyl\text{-}tetO}$*-ftsZ-halo*	This study
JE2 EzrA-sGFP spa-EzrA-HT	JE2 *ezrA::ezrA-sgfp* Δ*spa::*P$_{xyl\text{-}tetO}$*-ezrA-halo*	This study
JE2 EzrA-sGFP spa-FtsW-HT	JE2 *ezrA::ezrA-sgfp* Δ*spa::*P$_{xyl\text{-}tetO}$*-ftsW-halo*	This study
JE2 EzrA-sGFP spa-FtsW(W121A)-HT	JE2 *ezrA::ezrA-sgfp* Δ*spa::*P$_{xyl\text{-}tetO}$*-ftsW*$_{W121A}$*-halo*	This study
JE2 EzrA-sGFP spa-FtsW(D287A)-HT	JE2 *ezrA::ezrA-sgfp* Δ*spa::*P$_{xyl\text{-}tetO}$*-ftsW*$_{D287A}$*-halo*	This study
JE2 EzrA-sGFP spa-HT-DivIB(Δγ)	JE2 *ezrA::ezrA-sgfp* Δ*spa::*P$_{spac}$*-halo-divIB*$_{2\text{-}372}$	This study
JE2 EzrA-sGFP spa-iST-PBP1	JE2 *ezrA::ezrA-sgfp* Δ*spa::*P$_{xyl\text{-}tetO}$*-snap-pbp1*	This study
JE2 EzrA-sGFP spa-RodA-HT	JE2 *ezrA::ezrA-sgfp* Δ*spa::*P$_{spac}$*-rodA-halo*	This study

Table S5. Plasmids used in this study.

Name	Description	Reference
pSNAP-tag (T7)-2	*E. coli* expression vector encoding the Snap-tag protein; Amp^{r}	NEB
pIMAY-Z	*E. coli-S. aureus* shuttle vector with a thermosensitive origin of replication for Gram positive bacteria; Cm^{r}, *lacZ*	6
pIMAY-Z-ftsZ(T111A)	pIMAY-Z derivative containing *ftsZ*$_{T111A}$; Cm^{r}, *lacZ*	This study
pMAD	*E. coli-S. aureus* shuttle vector with a thermosensitive origin of replication for Gram positive bacteria; Amp^{r}, Ery^{r}, *lacZ*	7
pMAD-ezrAsgfp	pMAD derivative containing an *ezrA-sgfp* fusion and the downstream region of *ezrA;* Amp^{r}, Ery^{r}	5
pMAD-ftsWht	pMAD derivative containing an *ftsW-halo* fusion and the downstream region of *ftsW;* Amp^{r}, Ery^{r}	This study
pMAD-murJht	pMAD derivative containing a *murJ-halo* fusion and the downstream region of *murJ;* Amp^{r}, Ery^{r}	This study
pMAD-pbp4ht	pMAD derivative containing a *pbp4-halo* fusion and the downstream region of *pbp4;* Amp^{r}, Ery^{r}	This study
pMAD-gpsBht	pMAD derivative containing a *gpsB-halo* fusion and the downstream region of *gpsB;* Amp^{r}, Ery^{r}	This study
pCNX-ftsW(W121A)sgfp	pCNX derivative containing an *ftsW*$_{W121A}$*-sgfp* fusion; Amp^{r}, Kan^{r}	5
pCNX-ftsW(D287A)sgfp	pCNX derivative containing an *ftsW*$_{D287A}$*-sgfp* fusion; Amp^{r}, Kan^{r}	5
pBCB43	pMAD derivative with up- and downstream regions of the *spa* locus and *tetR*-P$_{xyl-tetO}$; Amp^{r}, Ery^{r}, *lacZ*	8
pBCB43-ftsZht	pBCB43 derivative containing an *ftsZ-halo* fusion; Amp^{r}, Ery^{r}, *lacZ*	This study
pBCB43-ezrAht	pBCB43 derivative containing an *ezrA-halo* fusion; Amp^{r}, Ery^{r}, *lacZ*	This study
pBCB43-ftsWht	pBCB43 derivative containing an *ftsW-halo* fusion; Amp^{r}, Ery^{r}, *lacZ*	This study
pBCB43-ftsW(W121A)ht	pBCB43 derivative containing an *ftsW*$_{W121A}$*-halo* fusion; Amp^{r}, Ery^{r}, *lacZ*	This study
pBCB43-ftsW(D287A)ht	pBCB43 derivative containing an *ftsW*$_{D287A}$*-halo* fusion; Amp^{r}, Ery^{r}, *lacZ*	This study
pBCB43-istpbp1	pBCB43 derivative containing an *isnap-pbp1* fusion; Amp^{r}, Ery^{r}, *lacZ*	This study
pBCB13	pMAD derivative with up- and downstream regions of the *spa* locus and *lacI*-P$_{spac}$; Amp^{r}, Ery^{r}, *lacZ*	9
pBCB13-Nht	pBCB13 derivative containing *halo-tag* for N-terminal protein fusions; Amp^{r}, Ery^{r}, *lacZ*	This study
pBCB13-htdivIB	pBCB13-htN derivative containing a *halo-divIB* fusion; Amp^{r}, Ery^{r}, *lacZ*	This study
pBCB13-htdivIB(Δγ)	pBCB13-htdivIB derivative encoding Halo-DivIB C-terminally truncated by 67 aa; Amp^{r}, Ery^{r}, *lacZ*	This study
pBCB13-htC	pBCB13 derivative containing *halo-tag* for C-terminal protein fusions; Amp^{r}, Ery^{r}, *lacZ*	This study
pBCB13-rodAht	pBCB13-htC derivative containing a *rodA-halo* fusion; Amp^{r}, Ery^{r}, *lacZ*	This study

Table S6. Oligonucleotides used in this study.

No.	Name	5'-3' sequence
3810	10aa linker-PBP1 for	GGCGGTTCTGGCGGAGGTGGCTCTGCGAAGCAAAAAATTAAAATTAAA
6700	COLftsZ_stop+58_rev	ATATCCCGGGCATCAGATATGTTATCTGATGATTTGT
6703	COLftsZ_-659_fwd	ATATGTCGACGATTCTGCTTCAGATCAAGATATCTTC
6713	ftsZ-RBS_fwd	ATATCCCGGGGGCCAATAAAACTAGGAGGAAATTTAA
6714	10aa-linker_rev	ATATCCCGGGAGTACTCGGCCGGTCGACAGAGCCACCTCCGCCAGAACCGCCTCCACC
6715	5aa-linker-C-halo-tag_fwd	ATATCCCGGGAGTACTCGGCCGGTCGACTCCTGCGGCGCCTCCGCGGAGATTGGAACTGGTTT
6716	C-halo-tag_rev	ATATCCCGGGTTAACCACTGATTTCTAAAGTAGATAACCATC
6726	COLftsZ-T111A_rev	AACGACTGGTGCTGCACCTGCACCAGTTCCGCCACCCATA
6727	COLftsZ-T111A_fwd	GCAGGTGCAGCACCAGTCGTT
6783	COLpbp4_-stop_rev	ATATGTCGACTTTTCTTTTTCTAAATAAACGATTGA
6785	COLmurJ_-stop_rev	ATATGTCGACTCGTAAAAACCTAACTCTACGTCTT
7031	C-HT_15aa_fwd	TTGGAAGGATCAGGACAAGGACCAGGATCTGGTCAAGGTTCTGGTGCGGAGATTGGAACTGGTTTCCCGT
7034	C-halo-tag_rev2	ATATCTCGAGCGGCCGTTAACCACTGATTTCTAAAGTAGATAACCATC
7139	COLezrA_st7_SmaI_fwd	ATATCCCGGGAAAAAATAAGGAGGAAAAAAAAATGGTGTTATATATCATTTTGGCAAT
7140	COLezrA-15aa_-stop_rev	AGATCCTGGTCCTTGTCCTGATCCTTCCAATTGCTTAATAACTTCTTCTTCAATA
7142	COLftsW-15aa_-stop_rev	AGATCCTGGTCCTTGTCCTGATCCTTCCAAATTAAATTGTCTTCTTATATCAAC
7191	COLftsZ+1_st7_fwd	ATATCCCGGGAAAAAATAAGGAGGAAAAAAAAATGTTAGAATTTGAACAAGGATTTAAT
7267	ftsZ-15aa_rev2	AGATCCTGGTCCTTGTCCTGATCCTTCCAAACGTCTTGTTCTTCTTGAACGTCTT
7369	HT_Kpn_rev	ATATGGTACCTTAACCACTGATTTCTAAAGTAG
7370	pbp4_800int_Eco_fwd	ATATGAATTCTTCGTCAATCCAACGGGTGCTG
7371	pbp4_800down_Kpn_fwd	ATATGGTACCAACATACTAAAAACGGACAAGTTGC
7372	pbp4_800down_Bam_rev	ATATGGATCCACCCAGCAGTAACGCACACGACAAT
7373	ftsW_800int_Eco_fwd	ATATGAATTCATGAACTTACAGGCATCTGAGT
7374	ftsW_800down_Kpn_fwd	ATATGGTACCAAAAATACTAGCCAATATTTAG
7375	ftsW_800down_Bam_rev	ATATGGATCCACGACGCGCAAATTGTTCATT
7376	murJ_800int_Bam_fwd	ATATGGATCCTACCTTCACAGTTACAAGATATATT
7377	murJ_800down_Kpn_fwd	ATATGGTACCTTAAGACGTAGAGTTAGGTT
7378	murJ_800down_Eco_rev	ATATGAATTCTCACGACTATCTTTACGTGTAACAAGT
7590	divIB_+4_Xho_fwd	ATATCTCGAGGATGATAAAACGAAGAACGATCAACA
7591	divIB_EagI_rev	ATATCGGCCGTTAATTATTCTTACTTGATTGTTTGT
7594	rodA_Eag_st7_fwd	ATATCGGCCGAAAAAATAAGGAGGAAAAAAAAATGAATTATTCATCTCGTCAACAGCCG
7595	rodA_-stop_Sal_rev	ATATGTCGACATTACTTTTTGGATGGTATAAATCGA
7597	gpsB_-stop_Sal_rev	ATATGTCGACTTTACCAAATACAGCTTTTTCTAAGTTT
7766	GA_gpsB-HT_1	GCATGCCATGGTACCCGGGAGCTCGAATTCATGGTTAAAACAGTTTATGTAACAG
7769	GA_gpsB-HT_4	TTGTATTTAGTAATTAACCACTGATTTCTAAAGTAG
7770	GA_gpsB-HT_5	ATCAGTGGTTAATTACTAAATACAAAAGTTTAACTGTC
7771	GA_gpsB-HT_6	TCCAGCCTCGCGTCGGGCGATATCGGATCCGTTCAACAATAGCTTTCTTAGTTATC
9171	divIB-372_EagI_rev	ATATCGGCCGTTATGATAATGATTGTGACATCTG
9247	ftsW_tetO-st3_fwd	TCTATCATTGATAGAGTCCCGGGAGCTCTCTATCATTGATAGAGTAAAAAAAAATAAGGAGGAAAATGAAGAATTTTAGAAGTATTTTACG
9631	iSNAP_st7_Sma_fwd	ATATCCCGGGAAAAAATAAGGAGGAAAAAAAAATGGATAAAAAAGGTTTGGAAATTTTTTTGGCTTCTGACAAAGATTGCGAAATGAAACG
9632	SNAP-10aa_rev	AGAGCCACCTCCGCCAGAACCGCCTCCACC*TCCCAGACCCGGTTTACCCAG*
9647	066-PBP1-stp-xbai	GGGCCCTCTAGATACGGCCGTTATTAGTCCGACTTATCCTTGTCAGTTTTAC

Underlined sequences correspond to restriction sites used for cloning.

Coding sequences for protein tags

halo-tag sequence (IDT)

ATGGCGGAGATTGGAACTGGTTTCCCGTTCGACCCTCACTACGTGGAGGTCTTAGGAGAACGTATGCACTACGTG
GACGTCGGTCCTCGTGATGGAACTCCAGTATTGTTCTTACATGGAAACCCTACTAGTTCATACGTGTGGCGAAAC
ATCATACCTCATGTGGCACCGACACACCGTTGTATCGCTCCTGATTTAATCGGAATGGGCAAAAGTGACAAGCCG
GACTTAGGTTATTTCTTTGACGATCACGTGCGATTTATGGATGCATTTATTGAGGCATTAGGATTAGAAGAAGTT
GTCTTGGTAATACATGATTGGGGCTCTGCATTGGGCTTCCACTGGGCGAAGAGAAACCCTGAGCGAGTAAAGGGC
ATCGCGTTCATGGAGTTTATTCGTCCAATCCCAACATGGGATGAATGGCCGGAATTTGCTAGAGAAACGTTCCAA
GCATTCCGTACTACTGACGTAGGCCGAAAGTTAATCATTGACCAGAACGTATTCATCGAGGGAACGTTACCTATG
GGAGTGGTTAGACCATTGACAGAAGTCGAGATGGACCATTACCGAGAGCCGTTTTTGAACCCAGTGGACAGAGAG
CCGTTATGGCGATTCCCTAACGAGTTACCTATTGCAGGTGAGCCGGCTAACATTGTCGCTTTGGTTGAAGAATAT
ATGGACTGGTTGCATCAATCACCGGTCCCAAAATTATTGTTTTGGGGAACACCTGGTGTGTTGATCCCACCGGCT
GAAGCTGCGCGTTTGGCGAAATCTTTGCCGAACTGTAAGGCGGTTGATATAGGTCCGGGATTAAACTTATTACAG
GAGGACAATCCTGACTTAATAGGCAGTGAGATCGCTAGATGGTTATCTACTTTAGAAATCAGTGGT

i-tag sequence [10]

ATGGATAAAAAAGGTTTGGAAATTTTTTTGGCTTCT

snap-tag sequence (NEB)

ATGGACAAAGATTGCGAAATGAAACGTACCACCCTGGATAGCCCGCTGGGCAAACTGGAACTGAGCGGCTGCGAA
CAGGGCCTGCATGAAATTAAACTGCTGGGTAAAGGCACCAGCGCGGCCGATGCGGTTGAAGTTCCGGCCCCGGCC
GCCGTGCTGGGTGGTCCGGAACCGCTGATGCAGGCGACCGCGTGGCTGAACGCGTATTTTCATCAGCCGGAAGCG
ATTGAAGAATTTCCGGTTCCGGCGCTGCATCATCCGGTGTTTCAGCAGGAGAGCTTTACCCGTCAGGTGCTGTGG
AAACTGCTGAAAGTGGTTAAATTTGGCGAAGTGATTAGCTATCAGCAGCTGGCGGCCCTGGCGGGTAATCCGGCG
GCCACCGCCGCCGTTAAAACCGCGCTGAGCGGTAACCCGGTGCCGATTCTGATTCCGTGCCATCGTGTGGTTAGC
TCTAGCGGTGCGGTTGGCGGTTATGAAGGTGGTCTGGCGGTGAAAGAGTGGCTGCTGGCCCATGAAGGTCATCGT
CTGGGTAAACCGGGTCTGGGA

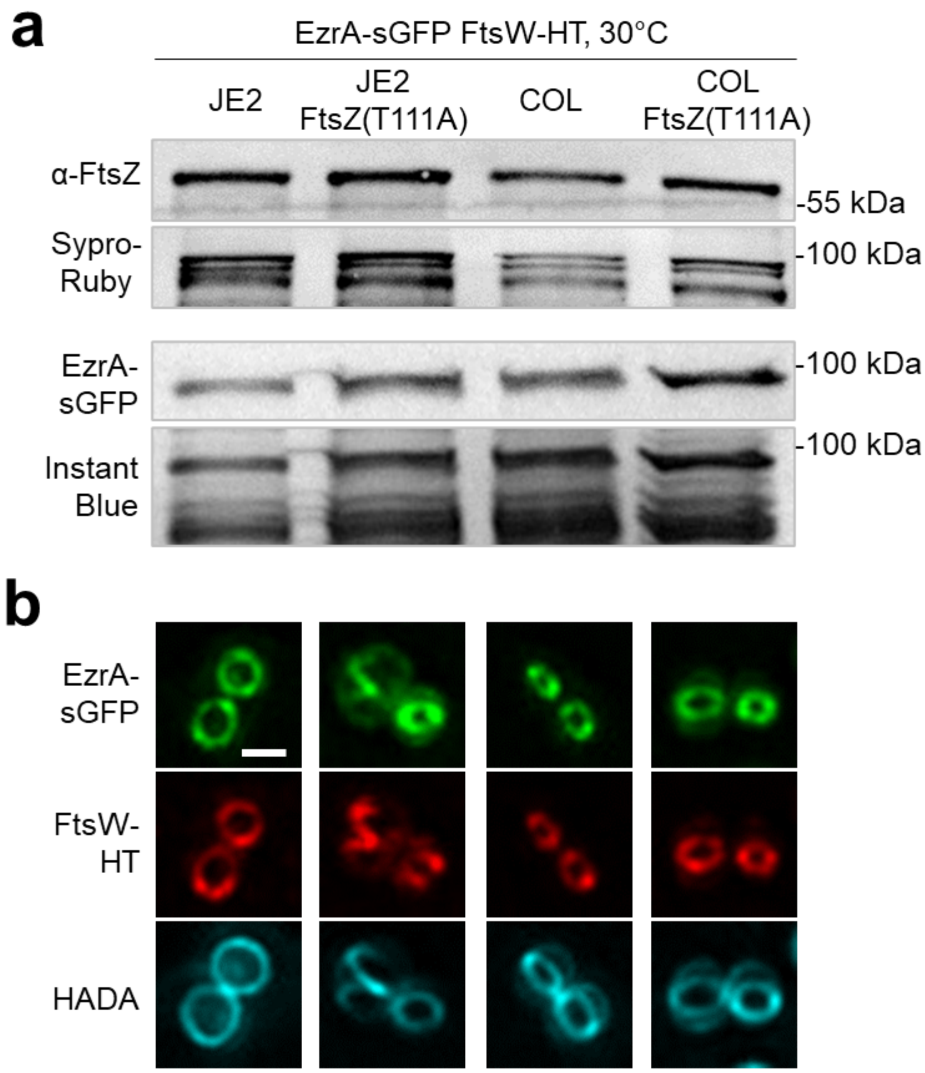

Figure S1. *S. aureus* FtsZ GTPase mutants maintain native FtsZ levels and incorporation of septal peptidoglycan. **a,** Western blot analysis and fluorescent protein gel of indicated strains grown in TSB rich medium at 30°C using anti-FtsZ antibody and green fluorescence detection for EzrA-sGFP, respectively. Total protein stains Sypro-Ruby and Instant Blue served as loading controls. Western blot analysis was performed in duplicate with similar results. Fluorescent protein gel analysis was performed once. **b,** Representative epifluorescence micrographs of strains indicated in panel **a** labelled with 500 nM JF549-HTL to visualize FtsW-HT and fluorescent D-amino acid HADA to visualize sites of nascent peptidoglycan synthesis. Images are representative from one of two biological repeats. Scale bar, 0.5 µm.

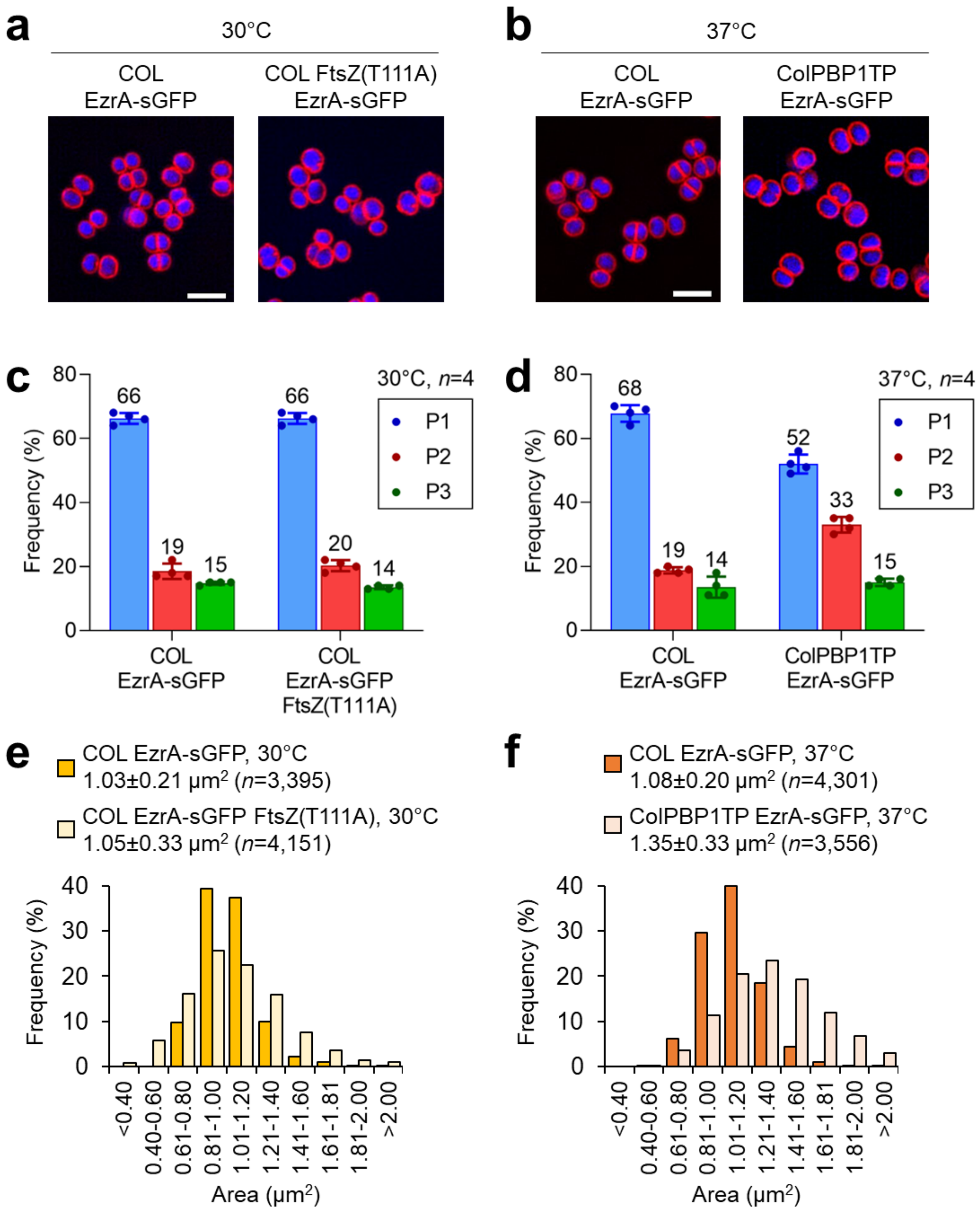

Figure S2. ***S. aureus*** **FtsZ GTPase and PBP1 TPase mutants are differentially affected in cell cycle progression and cell size. a,b,** Representative structured illumination micrographs of indicated strains grown to mid-exponential phase in TSB rich medium at indicated temperatures and labelled with the fluorescent dyes Nile red (membrane) and Hoechst 33342 (DNA). Images are representative from one of four biological repeats. Scale bars, 2 μm. **c-f,** Classification of cells into three cell-cycle phases (P1, no septum; P2, open septum; P3, closed septum) (**c**,**d**) and determination of cell area (**e**,**f**) for strains represented in panels **a** and **b**. Error bars represent the standard deviations for the mean from four biological replicates.

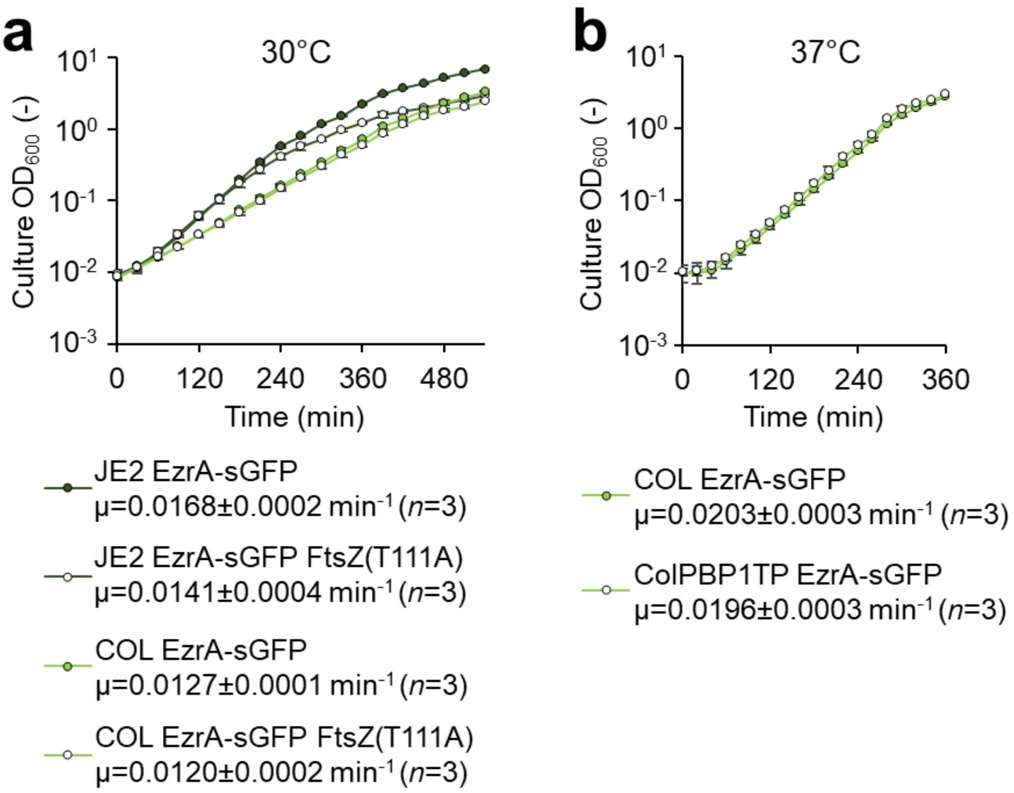

Figure S3. Growth curves of *S. aureus* FtsZ GTPase and PBP1 TPase mutant strains. Culture growth curves of indicated strains in TSB rich medium at the indicated temperatures. The OD_{600} was recorded every 30 min (**a**) or 20 min (**b**). Error bars represent the standard deviations for the mean from three biological replicates. Growth rate (μ) was calculated for cells in mid-exponential phase.

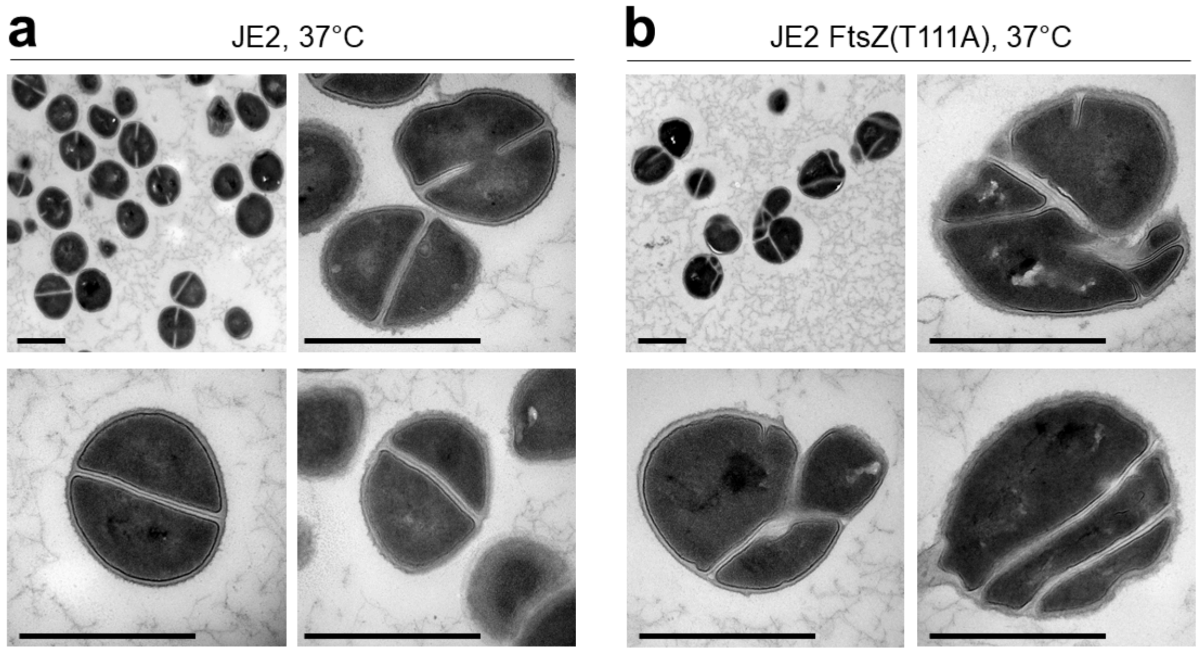

Figure S4. The FtsZ GTPase mutation T111A causes cell morphology defects in *S. aureus*. Transmission electron microscopy (TEM) images of JE2 wild-type and FtsZ(T111A) mutant derivative strains grown to mid-exponential phase in TSB rich medium at 37°C. TEM was performed once. Scale bars, 1 µm.

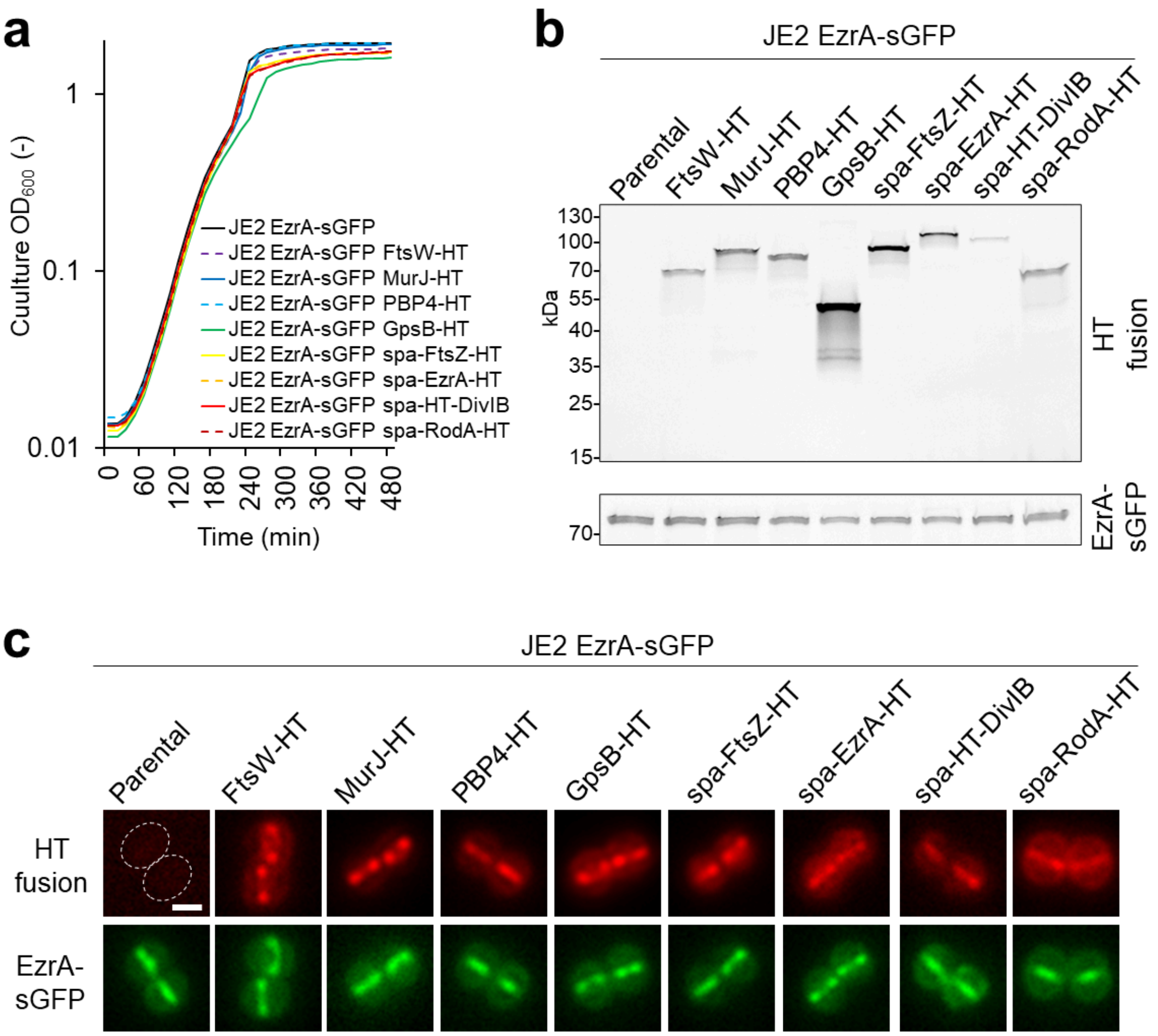

Figure S5. Halo-tag fusions to cell division and cell wall-related proteins produced in *S. aureus* are non-toxic and are enriched at mid-cell. a-c, Indicated strains were grown shaking in TSB rich medium at 37°C. Cells producing EzrA-HT or HT-DivIB were grown in the presence of 0.5 ng/ml Atc or 0.5 mM IPTG, respectively. **a,** Growth curves recorded in 96-well plate format and obtained from six biological replicates. **b,c,** Fluorescent protein gel (**b**) and representative epifluorescence micrographs (**c**) of cells grown to mid-exponential phase and labelled with 500 nM JF549-HTL. Dashed lines in the negative control indicate cell outlines inferred from the corresponding phase contrast image. Fluorescent protein gel and microscopy images are representative from one of two biological repeats. Theoretical molecular weights (in kDa): FtsW-HT, 79.6; MurJ-HT, 96.7; PBP4-HT, 83.0; GpsB-HT, 47.2; FtsZ-HT, 75.7; EzrA-HT, 101.0; HT-DivIB, 84.5; RodA-HT, 78.7; EzrA-sGFP, 93.6. Scale bar, 0.5 µm.

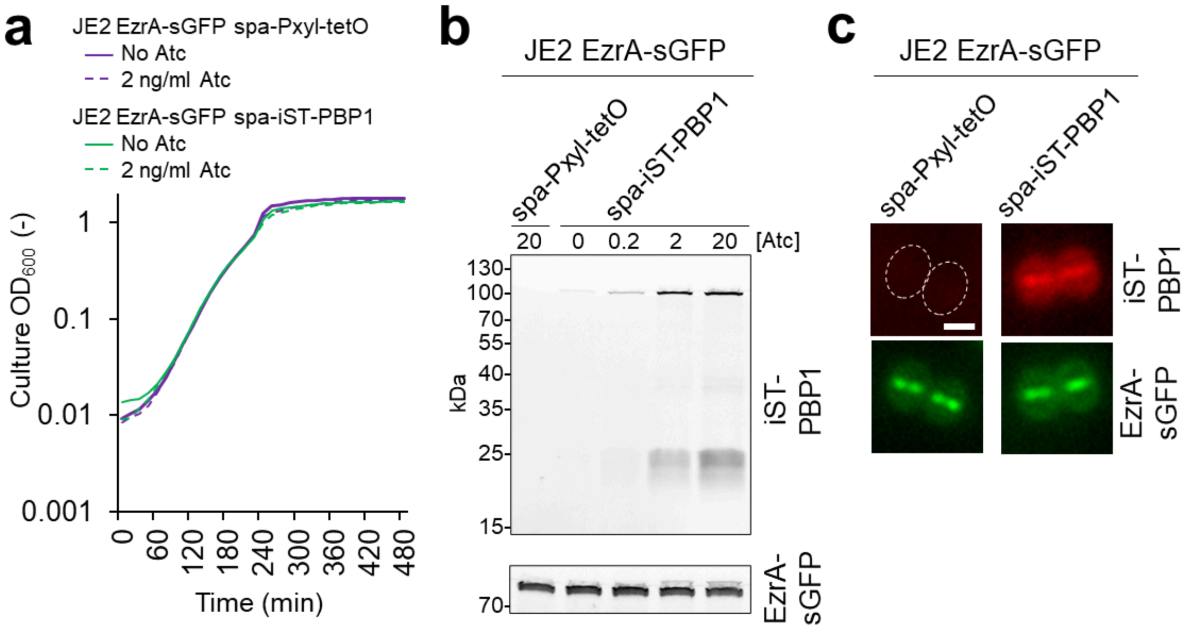

Figure S6. A Snap-tag fusion to PBP1 produced in *S. aureus* is non-toxic and is enriched at mid-cell. a-c, Strains indicated in panel **a** were grown shaking in TSB rich medium at 37°C and, where indicated, in the presence of Anhydrotetracycline (Atc) to induce gene expression from the heterologous xylose promoter containing *tetO* sites (P*xyl-tetO*). **a,** Growth curves recorded in 96-well plate format and obtained from six biological replicates. **b,c,** Fluorescent protein gel (**b**) and epifluorescence micrographs (**c**) of cells grown to mid-exponential phase and labelled with 500 nM JF549-cpSTL. Atc concentration is given in ng/ml. Dashed lines in the negative control indicate cell outlines inferred from the corresponding phase contrast image. Fluorescent protein gel analysis and microscopy imaging were performed once. The theoretical molecular weights of iST-PBP1 and EzrA-sGFP are 103.7 and 93.6 kDa, respectively. Scale bar, 0.5 µm.

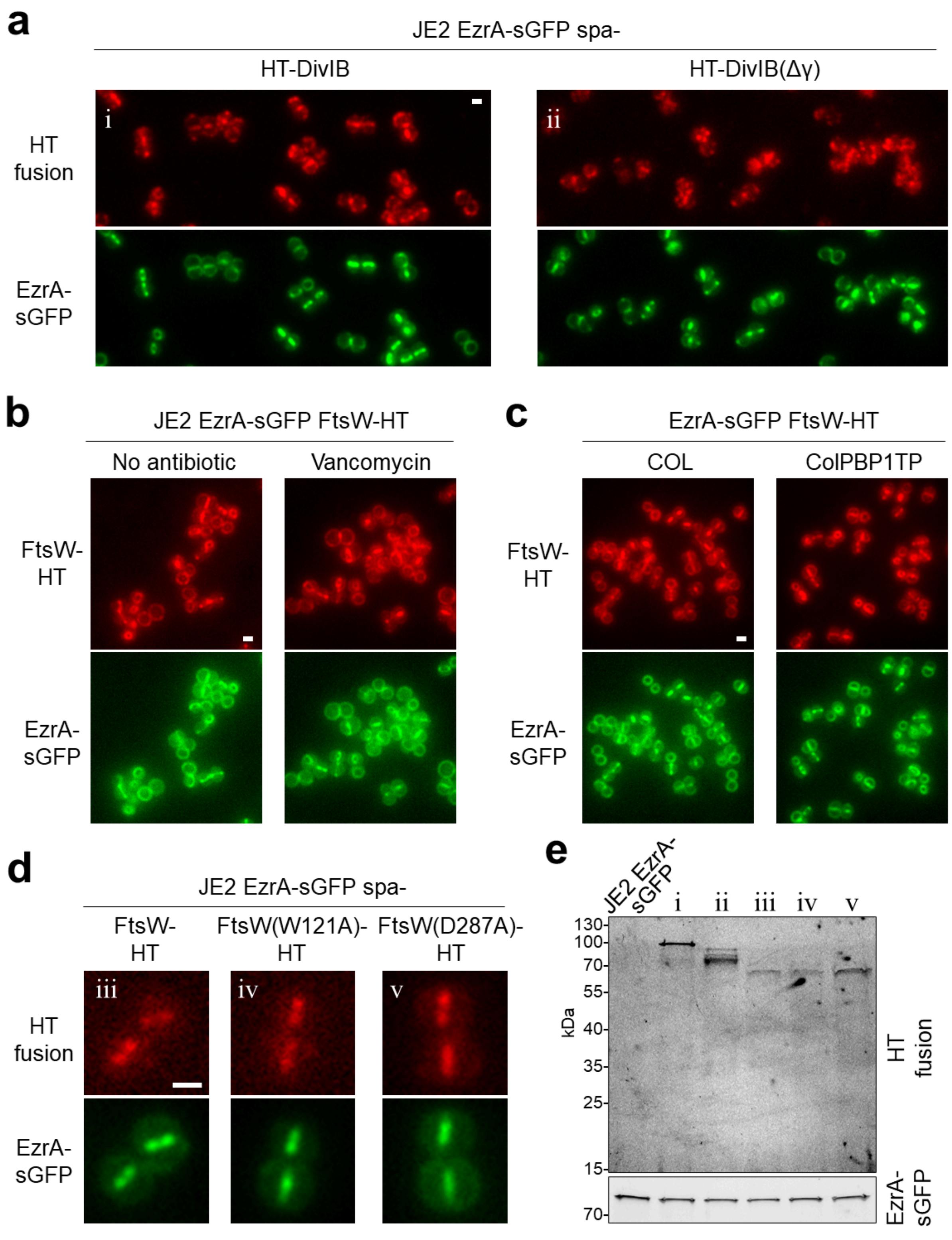

Figure S7. Localization of DivIB and FtsW variants in wild-type cells or in cells with perturbed peptidoglycan synthesis. a, Representative epifluorescence micrographs of cells producing, from the ectopic *spa* locus, the HT protein fused either to DivIB wild-type or to DivIB lacking its C-terminal γ domain. Indicated strains were grown in TSB rich medium supplemented with 0.5 mM IPTG at 37°C. Cells were labelled with 500 nM JF549-HTL after reaching mid-exponential growth phase. **b,c,** Representative epifluorescence micrographs of cells producing FtsW-HT from its

native genomic locus in indicated genetic backgrounds. Exponentially growing cells were incubated with 500 nM JF549-HTL and with or without 2 μg/ml vancomycin for 20 min at 37°C. **d,** Representative epifluorescence micrographs of cells producing FtsW-HT wild-type or active-site mutant derivatives W121A and D287A from the ectopic *spa* locus. Indicated strains were grown in TSB rich medium supplemented with 0.2 ng/ml Atc at 37°C. Cells were labelled with 500 nM JF549-HTL after reaching mid-exponential growth phase. **e,** Fluorescent protein gel for strains indicated by i to v in panels **a** and **d**. Theoretical molecular weights (in kDa): HT-DivIB, 84.5; HT-DivIB(Δγ), 77.1; FtsW-HT, 79.6; EzrA-sGFP, 93.6. Fluorescent protein gel and microscopy images are representative from one of two biological repeats. Scale bars, 0.5 μm.

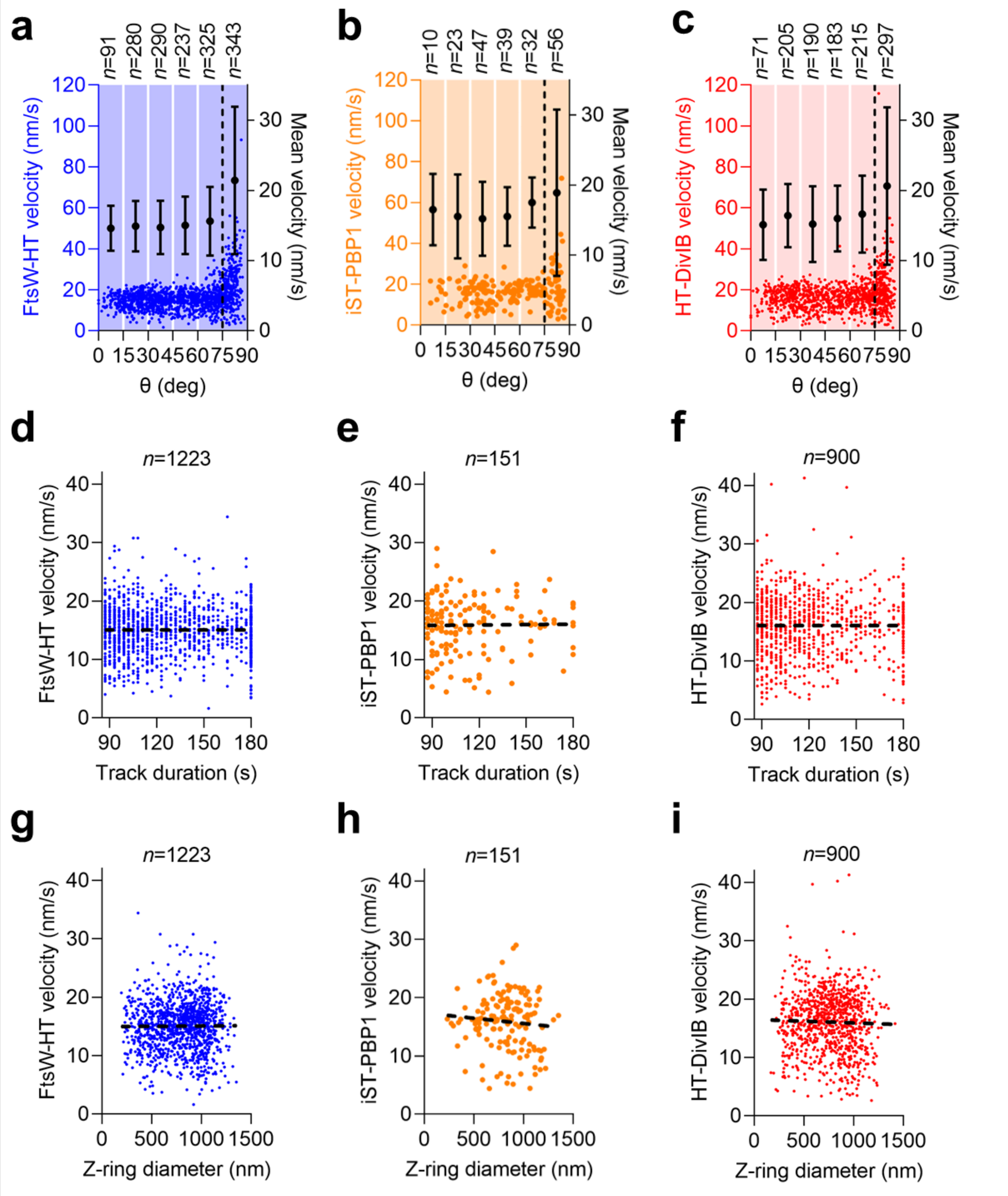

Figure S8. FtsW, PBP1 and DivIB average velocities do not correlate with track duration or cell division stage. FtsW-HT (**a,d,g**), iST-PBP1 (**b,e,h**) and HT-DivIB (**c,f,i**) single-molecule velocity as a function of the angle between the imaging and cell division planes (Θ) (**a-c**), track duration (**d-f**) and EzrA-sGFP ring diameter (**g-i**). Each point in a graph corresponds to the calculated average velocity of a trajectory. JE2 EzrA-sGFP derivative strains were grown in TSB rich medium at 37°C and in the presence of 0.5 mM IPTG (HT-DivIB) or 2 ng/ml Atc (iST-PBP1). The cut-off for Θ at 75° (**a-c**) and simple linear regressions (**d-i**) are indicated by black dashed lines. Black points and error bars represent the means and standard deviations for average track velocities in the corresponding range of Θ (indicated by white vertical lines). n, number of trajectories obtained for cells examined over at least six independent experiments.

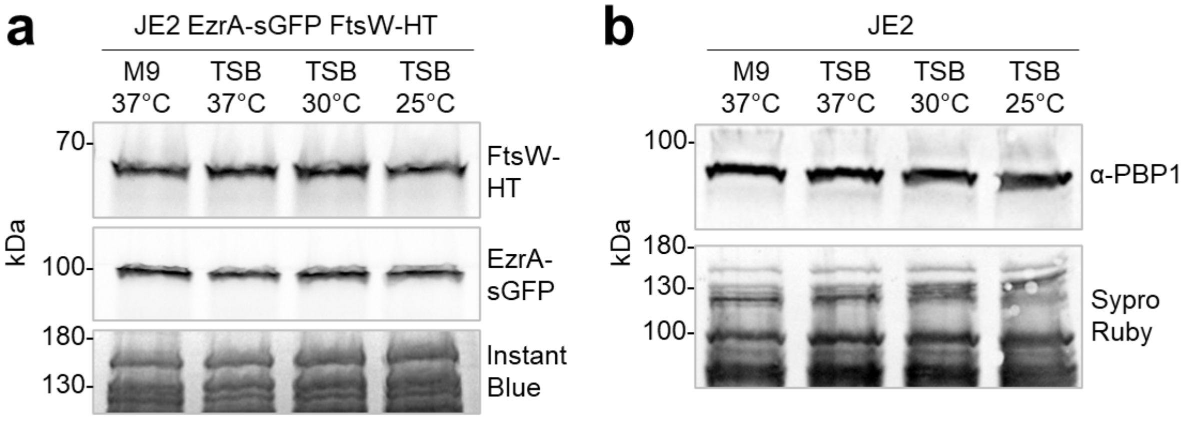

Figure S9. Levels of cell division proteins are similar in fast- and slow-growing cells. a, Fluorescent protein gel of the strain JE2 EzrA-sGFP FtsW-HT grown in M9 minimal medium at 37°C or in TSB rich medium at 37, 30 and 25°C. After reaching mid-exponential phase, cells were labelled with 500 nM JF549-HTL for 20 min. EzrA-sGFP and FtsW-HT were visualized using green and red fluorescence detection, respectively. **b,** Western blot analysis of the JE2 wild-type strain grown in the same conditions as cells in panel **a** using anti-PBP1 antibody. Total protein stains Instant Blue and Sypro-Ruby served as loading controls. Specific cell growth rates determined in the identical growth conditions are shown in Figure 4. Immunoblot and fluorescent protein gel analyses were performed once.

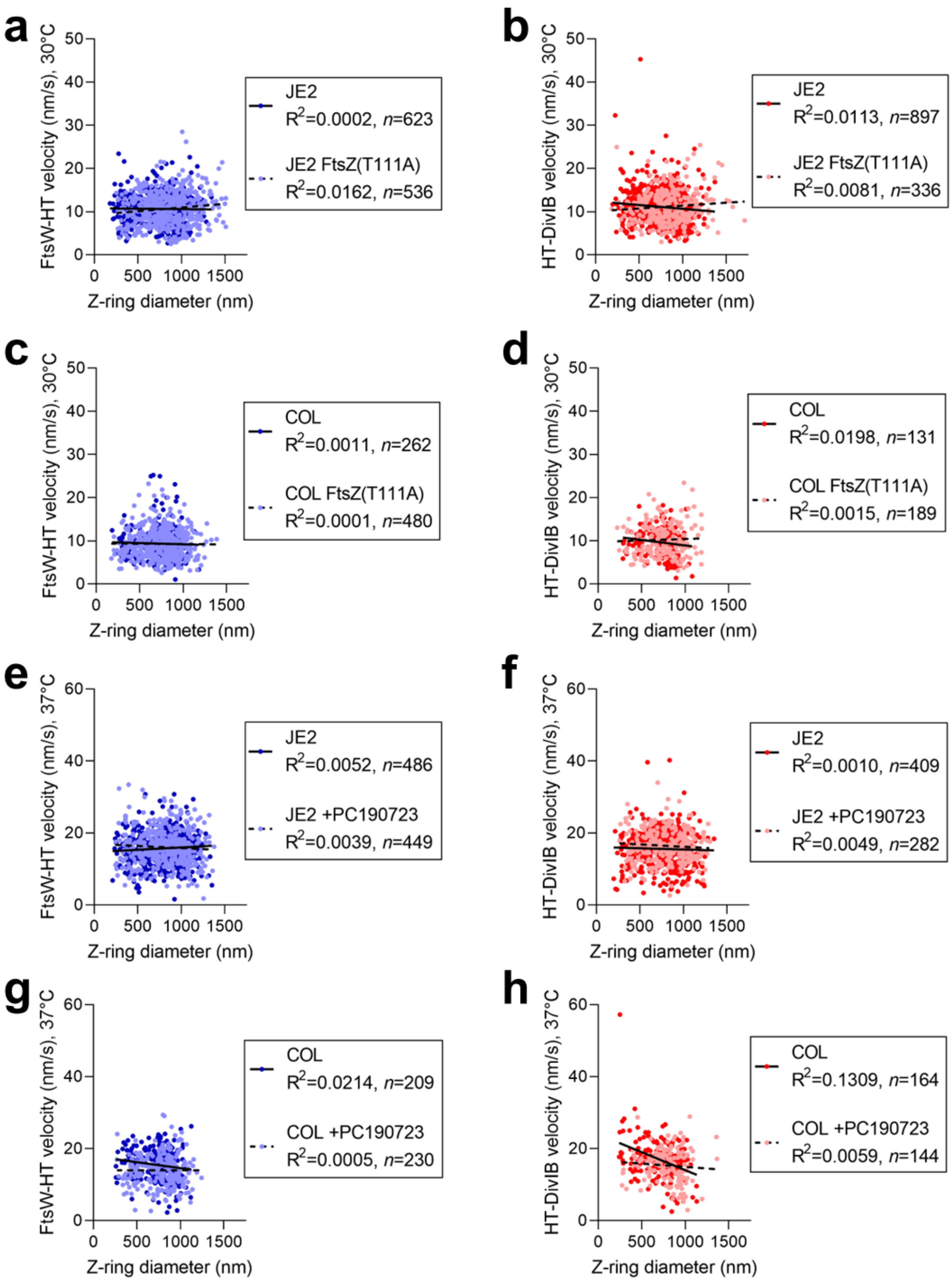

Figure S10. FtsW and DivIB average velocities remain unchanged when FtsZ treadmilling is impaired at any stage of cell division. FtsW-HT (**a,c,e,g**) and HT-DivIB (**b,d,f,h**) single-molecule velocity as a function of EzrA-sGFP ring diameter in cells containing FtsZ mutation T111A or treated with 5 µg/ml PC190723 for 2 min. Cells of the indicated strains were grown in TSB rich medium at 30°C (**a-d**) or at 37°C (**e-h**), and in the presence of 0.5 mM IPTG (**b,d,f,h**). Each point in a graph corresponds to the calculated average velocity of a trajectory. Simple linear regressions are indicated as continuous and dashed lines. n, number of trajectories obtained for cells examined over three independent experiments.

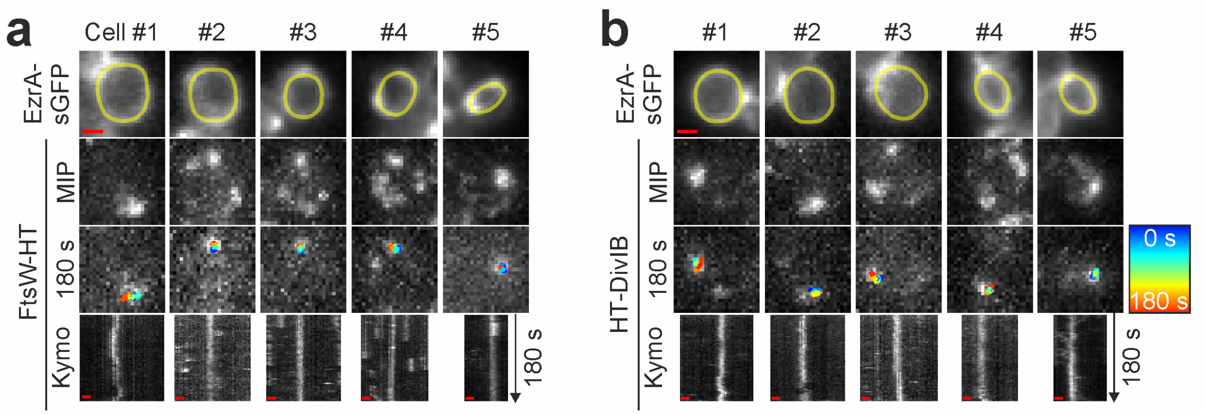

Figure S11. Directional movement of FtsW and DivIB is stopped by vancomycin. a,b, Representative epifluorescence micrographs of JE2 EzrA-sGFP producing FtsW-HT (**a**) or HT-DivIB (**b**). Cells producing HT-DivIB were grown in the presence of 0.5 mM IPTG to induce gene expression from the ectopic *spa* locus. Cells were treated with 2 μg/ml vancomycin for 20 min at 37°C and sparsely labelled with the fluorescent ligand JF549-HTL to visualize single molecules of FtsW-HT and HT-DivIB. Five independent cells are shown in each panel. Single-molecule images acquired in the last frame of a 180-s time series are overlayed with tracks, where blue (0 s) to red (180 s) indicates trajectory time. Space-time kymographs were generated by extracting fluorescence intensity values from FtsW-HT and HT-DivIB images along yellow lines drawn over corresponding EzrA-sGFP rings acquired with the last frame of each time series. MIP, maximum intensity projection. Images are representative from one of three independent experiments. Scale bars, 0.5 μm.

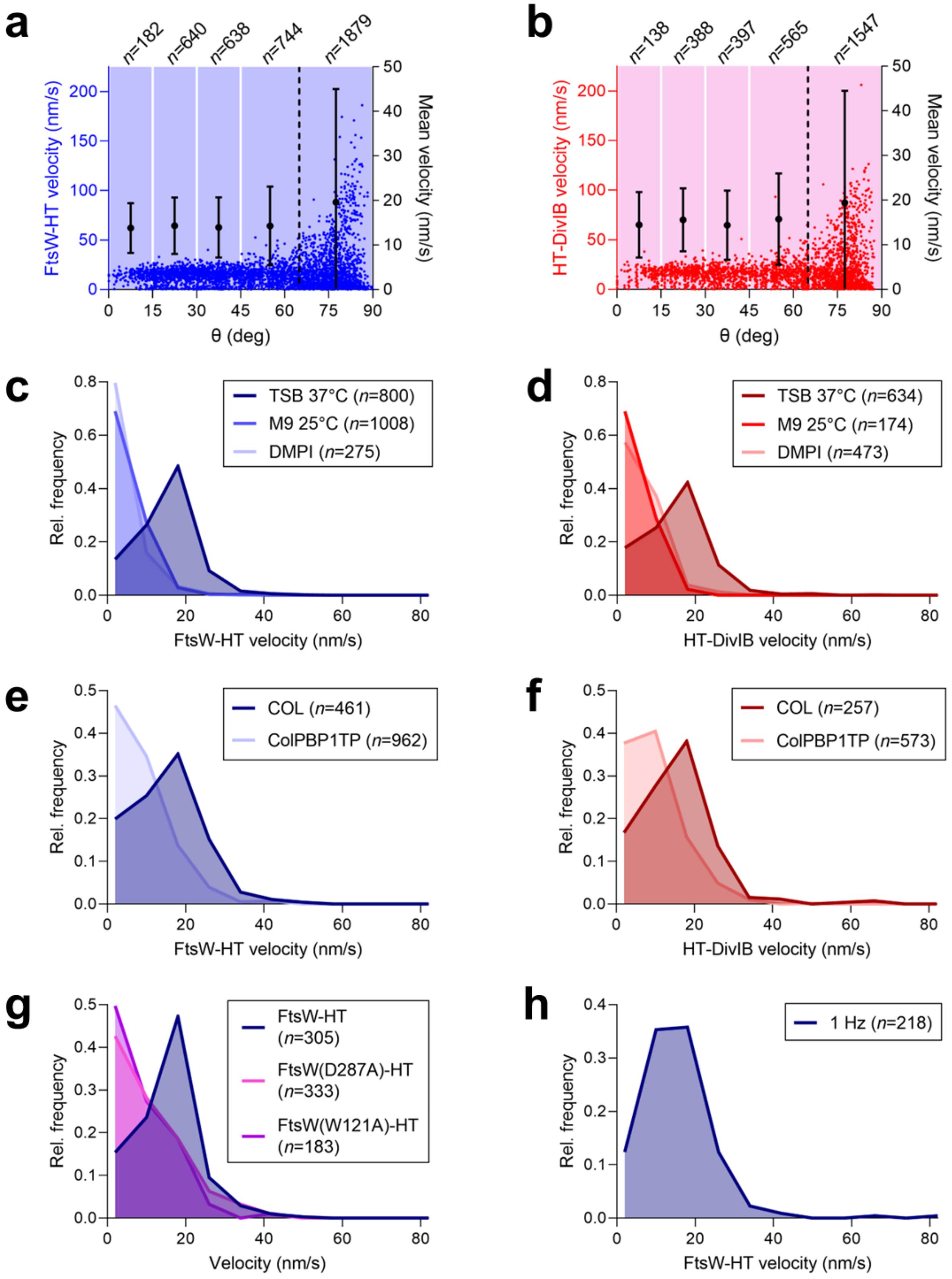

Figure S12. FtsW and DivIB sectional velocities show a unimodal distribution. a,b, FtsW-HT (**a**) and HT-DivIB (**b**) single-molecule velocity as a function of the angle between the imaging and cell division planes (Θ). Each point in a graph corresponds to the calculated velocity in each section of a trajectory. The cut-off for Θ at 65° is indicated by black dashed lines. Black points and error bars represent the means and

standard deviations for velocities obtained in each section of a trajectory in the corresponding range of Θ (indicated by white vertical lines). Data was obtained for JE2 EzrA-sGFP derivative strains grown in TSB rich medium at 37°C and corresponds to average velocities shown in Supplementary Figure S8a,c. **c-h,** Histograms depicting the velocity distribution for FtsW-HT (**c,e,g,h**) and HT-DivIB (**d,f**) determined in JE2 EzrA-sGFP and COL EzrA-sGFP derivative strains grown in the indicated conditions. Each data point in the histogram corresponds to the velocity measured in each section of a trajectory. Bin width, 8. Center of first/last bin, 2/82. Unless otherwise specified, the frame rate for image acquisition was 0.33 Hz. n, number of trajectory sections obtained for cells examined over three independent experiments.

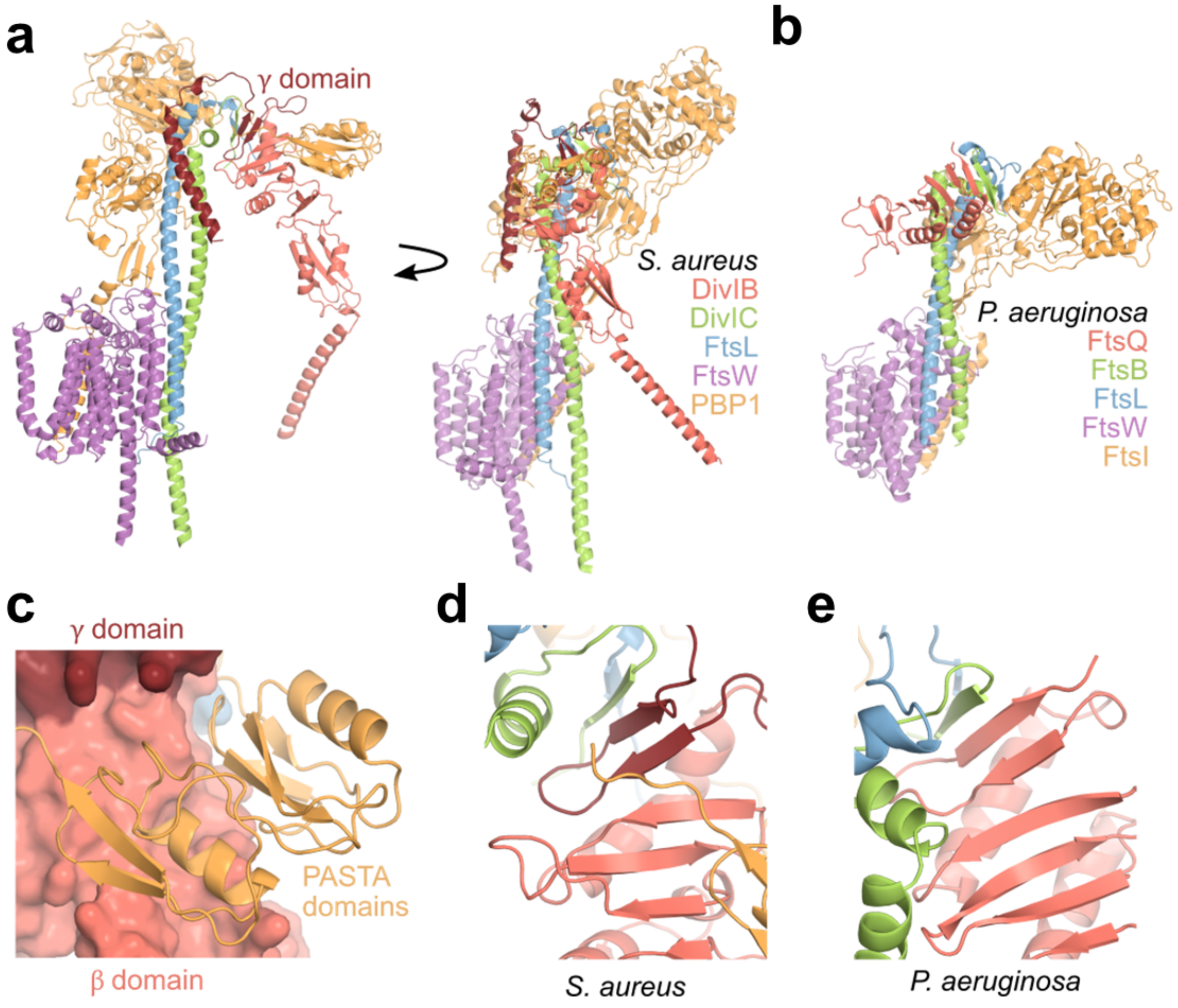

Figure S13. Structure prediction of a putative pentameric complex composed of the *S. aureus* proteins FtsW, PBP1, DivIB, DivIC and FtsL. a, Alphafold-multimer model of the complex formed by FtsW (purple), PBP1 (orange), DivIB (red), DivIC (green) and FtsL (blue) from *S. aureus*. DivIB γ-domain residues 373-439 are shown in a darker red colour. Terminal residues with low local prediction confidence are not shown (pLDDT below 50), except for DivIB C-terminal residues and PBP1 residues 590–594 between the TPase and PASTA domains. **b,** Cryo-EM structure of the orthologous *P. aeruginosa* complex (PDB 8BH1) aligned to FtsW in the *S. aureus* complex shown on the right in panel **a**. A comparison between these two structures shows a similar predicted arrangement of domains and a similar tilt of the TPase domain to that observed between predicted and experimental structures of the divisome complex[11,12]. **c,** C-terminal PASTA domains in PBP1 are predicted to interact primarily with the DivIB β domain. **d,e,** Comparison of the local structures after alignment to γ-domain residues shows that the *S. aureus* γ domain is predicted to contribute two strands to a β sheet linking DivIB, DivIC, and FtsL, as was observed experimentally for *P. aeruginosa*. Relative orientations of DivIC and FtsL helices differ in the *S. aureus* prediction.

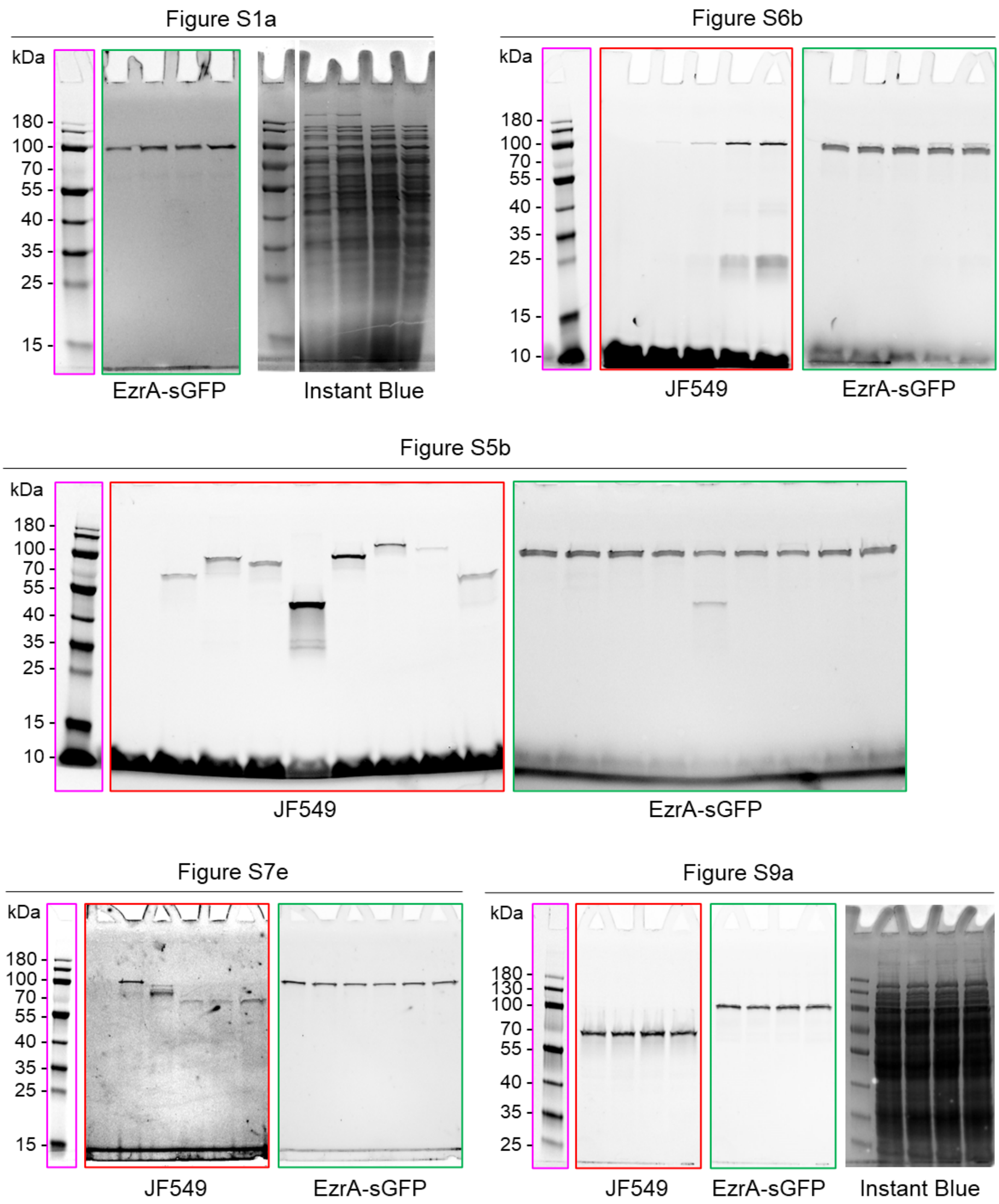

Figure S14. Uncropped images of gels shown in Supplementary Figures 1a, 5b, 6b, 7e and 9a. JF549-labelled HT and ST protein fusions (outlined in red), EzrA-sGFP (outlined in green) and molecular weight markers (outlined in magenta) visualized by red, green and far-red fluorescence detections, respectively, and total proteins visualized by post-staining with Instant Blue Coomassie.

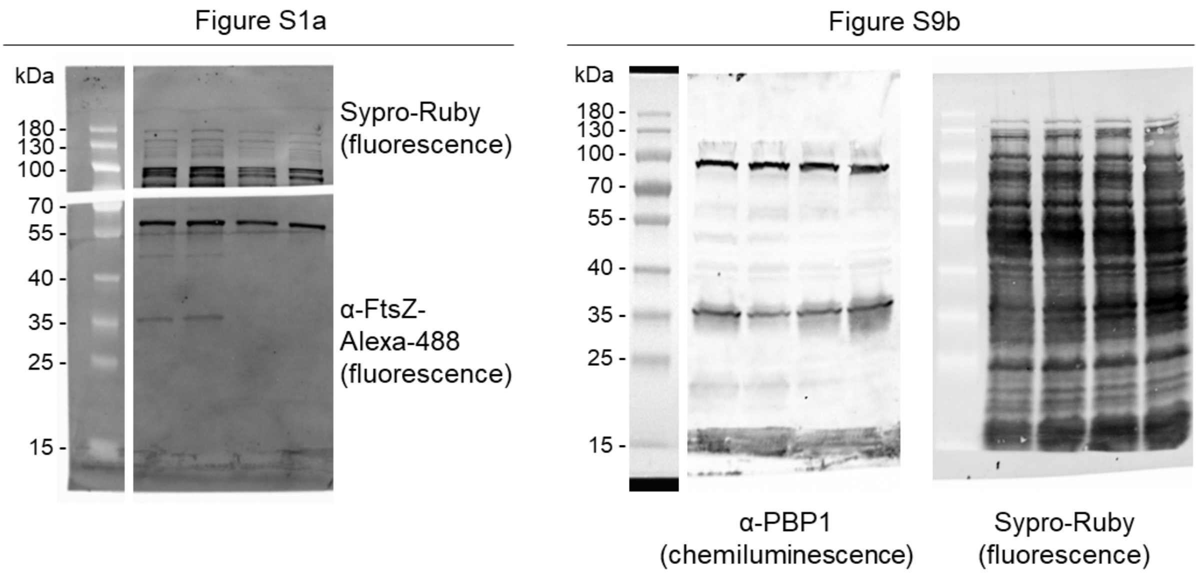

Figure S15. Uncropped images of blotted membranes shown in Supplementary Figures 1a and 9b. FtsZ and PBP1 visualized by green fluorescence and chemiluminescence detections, respectively, and total proteins visualized by staining with Sypro-Ruby.

References

1 Monk, I. R., Shah, I. M., Xu, M., Tan, M. W. & Foster, T. J. Transforming the untransformable: application of direct transformation to manipulate genetically *Staphylococcus aureus* and *Staphylococcus epidermidis*. *mBio* **3**, e00277-00211, doi:10.1128/mBio.00277-11 (2012).

2 Nair, D. *et al.* Whole-genome sequencing of *Staphylococcus aureus* strain RN4220, a key laboratory strain used in virulence research, identifies mutations that affect not only virulence factors but also the fitness of the strain. *J Bacteriol* **193**, 2332-2335, doi:10.1128/jb.00027-11 (2011).

3 Fey, P. D. *et al.* A genetic resource for rapid and comprehensive phenotype screening of nonessential *Staphylococcus aureus* genes. *mBio* **4**, e00537-00512, doi:10.1128/mBio.00537-12 (2013).

4 Saraiva, B. M. *et al.* Reassessment of the distinctive geometry of *Staphylococcus aureus* cell division. *Nat Commun* **11**, 4097, doi:10.1038/s41467-020-17940-9 (2020).

5 Reichmann, N. T. *et al.* SEDS-bPBP pairs direct lateral and septal peptidoglycan synthesis in *Staphylococcus aureus*. *Nat Microbiol* **4**, 1368-1377, doi:10.1038/s41564-019-0437-2 (2019).

6 Monk, I. R., Tree, J. J., Howden, B. P., Stinear, T. P. & Foster, T. J. Complete bypass of restriction systems for major *Staphylococcus aureus* lineages. *mBio* **6**, e00308-00315, doi:10.1128/mBio.00308-15 (2015).

7 Arnaud, M., Chastanet, A. & Debarbouille, M. New vector for efficient allelic replacement in naturally nontransformable, low-GC-content, gram-positive bacteria. *Appl. Environ. Microbiol.* **70**, 6887-6891, doi:10.1128/AEM.70.11.6887-6891.2004 (2004).

8 Reed, P. *et al.* A CRISPRi-based genetic resource to study essential *Staphylococcus aureus* genes. *mBio*, doi:10.1128/mbio.02773-23 (2023).

9 Pereira, P. M., Veiga, H., Jorge, A. M. & Pinho, M. G. Fluorescent reporters for studies of cellular localization of proteins in *Staphylococcus aureus*. *Appl. Environ. Microbiol.* **76**, 4346-4353, doi:10.1128/AEM.00359-10 (2010).

10 Catalão, M. J., Figueiredo, J., Henriques, M. X., Gomes, J. P. & Filipe, S. R. Optimization of fluorescent tools for cell biology studies in Gram-positive bacteria. *PLoS ONE* **9**, e113796, doi:10.1371/journal.pone.0113796 (2014).

11 Britton, B. M. *et al.* Conformational changes in the essential *E. coli* septal cell wall synthesis complex suggest an activation mechanism. *Nat Commun* **14**, 4585, doi:10.1038/s41467-023-39921-4 (2023).

12 Käshammer, L. *et al.* Cryo-EM structure of the bacterial divisome core complex and antibiotic target FtsWIQBL. *Nat Microbiol*, doi:10.1038/s41564-023-01368-0 (2023).
