# Supplementary material for: Cell constriction requires processive septal peptidoglycan synthase movement independent of FtsZ treadmilling in Staphylococcus aureus
Source: Nat Microbiol. 2024 Mar 13;9(4):1049–63. doi: 10.1038/s41564-024-01629-6 (PMC10994846; doi:10.1038/s41564-024-01629-6)
Supplement: Supplementary file 2 — Reporting Summary [file 41564_2024_1629_MOESM2_ESM.pdf]

nature portfolio

Corresponding author(s): Simon Schäper, Mariana G. Pinho

Last updated by author(s): Jan 11, 2024

Reporting Summary

Nature Portfolio wishes to improve the reproducibility of the work that we publish. This form provides structure for consistency and transparency in reporting. For further information on Nature Portfolio policies, see our Editorial Policies and the Editorial Policy Checklist.

Statistics

For all statistical analyses, confirm that the following items are present in the figure legend, table legend, main text, or Methods section.

n/a	Confirmed	
☐	☒	The exact sample size (*n*) for each experimental group/condition, given as a discrete number and unit of measurement
☐	☒	A statement on whether measurements were taken from distinct samples or whether the same sample was measured repeatedly
☐	☒	The statistical test(s) used AND whether they are one- or two-sided<br>*Only common tests should be described solely by name; describe more complex techniques in the Methods section.*
☒	☐	A description of all covariates tested
☒	☐	A description of any assumptions or corrections, such as tests of normality and adjustment for multiple comparisons
☐	☒	A full description of the statistical parameters including central tendency (e.g. means) or other basic estimates (e.g. regression coefficient) AND variation (e.g. standard deviation) or associated estimates of uncertainty (e.g. confidence intervals)
☐	☒	For null hypothesis testing, the test statistic (e.g. *F*, *t*, *r*) with confidence intervals, effect sizes, degrees of freedom and *P* value noted<br>*Give P values as exact values whenever suitable.*
☒	☐	For Bayesian analysis, information on the choice of priors and Markov chain Monte Carlo settings
☒	☐	For hierarchical and complex designs, identification of the appropriate level for tests and full reporting of outcomes
☒	☐	Estimates of effect sizes (e.g. Cohen's *d*, Pearson's *r*), indicating how they were calculated

Our web collection on statistics for biologists contains articles on many of the points above.

Software and code

Policy information about availability of computer code

Data collection	Image data was collected using the softwares AcquireSR v4.4 (GE Healthcare) and ZEN (Zeiss; blue edition v2.0.0.0 for acquiring epifluorescence images; black edition v8.1.0.484 for acquiring and reconstructing SIM images).
Data analysis	Image processing was done in the softwares SoftWorX v7.2.1 (GE Healthcare) (channel alignment, maximum intensity projection, deconvolution, SIM image reconstruction) and ImageJ/Fiji v1.53 (Schindelin et al. 2012) (maximum intensity projection, scale bars, lookup tables, movie montages, kymographs, slope measurements, thresholding). The ImageJ/Fiji plugin NanoJ v2.1RC1 (Laine et al. 2019) was used to perform drift correction for EzrA-sGFP time-lapse images. Spot detection and linking was performed in the ImageJ/Fiji plugin TrackMate v.7.2.0 (Tinevez et al. 2017) using parameters indicated in the methods section. The in-house developed software eHooke (Saraiva et al. 2021) was used to determine cell-cycle phases and measure the area of cells. Post-processing and analysis of single-molecule tracking data was done in the in-house developed software AureusSpeedTracker. The custom code is available at https://github.com/BacterialCellBiologyLab/AureusSpeedTracker . GraphPad Prism v9.1.0 was used to calculate means, standard deviations and linear regressions, to generate histograms and to perform statistical analysis (two-tailed Mann-Whitney U-test).

For manuscripts utilizing custom algorithms or software that are central to the research but not yet described in published literature, software must be made available to editors and reviewers. We strongly encourage code deposition in a community repository (e.g. GitHub). See the Nature Portfolio guidelines for submitting code & software for further information.

Data

Policy information about availability of data

All manuscripts must include a data availability statement. This statement should provide the following information, where applicable:

- Accession codes, unique identifiers, or web links for publicly available datasets
- A description of any restrictions on data availability
- For clinical datasets or third party data, please ensure that the statement adheres to our policy

Raw data for all figures are available from the corresponding authors upon reasonable request. Source data for Figures and Supplementary Figures are provided with the manuscript. Unprocessed imaging data for Supplementary Videos 1-5, 7 and 8 are available at https://figshare.com/projects/SaureusDivisomeDynamics/191457 .

Research involving human participants, their data, or biological material

Policy information about studies with human participants or human data. See also policy information about sex, gender (identity/presentation), and sexual orientation and race, ethnicity and racism.

Reporting on sex and gender	Research does not involve human participants, their data, or biological material.
Reporting on race, ethnicity, or other socially relevant groupings	Research does not involve human participants, their data, or biological material.
Population characteristics	Research does not involve human participants, their data, or biological material.
Recruitment	Research does not involve human participants, their data, or biological material.
Ethics oversight	Research does not involve human participants, their data, or biological material.

Note that full information on the approval of the study protocol must also be provided in the manuscript.

Field-specific reporting

Please select the one below that is the best fit for your research. If you are not sure, read the appropriate sections before making your selection.

☒ Life sciences ☐ Behavioural & social sciences ☐ Ecological, evolutionary & environmental sciences

For a reference copy of the document with all sections, see nature.com/documents/nr-reporting-summary-flat.pdf

Life sciences study design

All studies must disclose on these points even when the disclosure is negative.

Sample size	No sample size calculation was made or statistical method applied to assess sample size. Sample sizes varied from 5 to 4301 depending on the experiment. For most experiments, data from more than 100 cells were analysed, which is usual in bacterial microscopy. In some experiments, the sample size was smaller due to a very laborious image analysis by hand (i.e. cell constriction rate) or a very low number of quantifiable cells in a field-of-view (e.g. cells that showed directionally moving molecules of FtsW(W121A)-HT or displayed EzrA-sGFP patches in the presence of FtsZ-T111A or after PC190723 treatment). Specific sample sizes are reported in the Figures, Figure legends and Supplementary Tables 1, 2 & 3.
Data exclusions	All filtering processes applied uniformly to all samples are described in the methods section and in the legends of Supplementary Tables 2 & 3. Cells out of focus or inaccurately segmented were excluded from the analysis. Cells were excluded from the analysis when they did not exhibit single molecules moving directionally, presented aberrantly shaped Z-rings (FtsZ-T111A mutant in the JE2 EzrA-sGFP background), or single-molecule tracks did not overlap with EzrA-sGFP rings. FtsZ treadmilling speed was not determined for cells that did not show a fluorescent patch of EzrA-sGFP. Septum constriction rate was determined for cells randomly selected in a field of view that showed visible constriction over a minimum of five consecutive time frames and that completed septum constriction during the observation period (except for DMPI-treated cells).
Replication	Single-molecule experiments in identical conditions were performed in triplicate on the same day. Single-molecule imaging in the standard growth condition (TSB rich medium at 37°C) and of specific protein fusions (FtsW-HT, HT-DivIB, iST-PBP1) was performed for at least six biological replicates on different days with similar results. A single replicate was analysed for protein fusions with no detectable directional movement (Supplementary Table 2). Septum constriction and FtsZ treadmilling rates were obtained for two biological replicates on different days. To determine growth rates, growth curves in identical conditions were recorded in triplicate on the same day. Cell-cycle phase classifications and cell area measurements were performed in four repeats. All attempts at replication were successful. Immunoblots shown in Supplementary Figures 1a & 9b and fluorescent protein gels shown in Supplementary Figures 1a, 5b, 6b, 7e & 9a were performed either once or twice as indicated in the figure legends.
Randomization	Samples were not allocated to groups.

Blinding	Blinding was not relevant as samples were not allocated to groups.

Reporting for specific materials, systems and methods

We require information from authors about some types of materials, experimental systems and methods used in many studies. Here, indicate whether each material, system or method listed is relevant to your study. If you are not sure if a list item applies to your research, read the appropriate section before selecting a response.

Materials & experimental systems

n/a	Involved in the study
☐	☒ Antibodies
☒	☐ Eukaryotic cell lines
☒	☐ Palaeontology and archaeology
☒	☐ Animals and other organisms
☒	☐ Clinical data
☒	☐ Dual use research of concern
☒	☐ Plants

Methods

n/a	Involved in the study
☒	☐ ChIP-seq
☒	☐ Flow cytometry
☒	☐ MRI-based neuroimaging

Antibodies

Antibodies used	Anti-FtsZ antibody (provided by Elizabeth Harry, University of Technology, Sydney) raised against B. subtilis FtsZ in sheep and diluted 1:2,000. Anti-PBP1 antibody (non-commercial, Eurogentec) raised against S. aureus PBP1 in rabbit and diluted 1:1,000. Alexa488 anti-sheep antibody (Invitrogen) produced in donkey and diluted 1:50,000. Horseradish peroxidase (HRP) anti-rabbit antibody (GE Healthcare) produced in donkey and diluted 1:50,000.
Validation	Anti-FtsZ and anti-PBP1 antibodies against whole cell extracts have been published (Pereira et al. 2016 (mBio) and Reed et al. 2015 (Plos Pathogens), respectively).

Plants

Seed stocks	Research does not involve plants.
Novel plant genotypes	Research does not involve plants.
Authentication	Research does not involve plants.
